# Genetic Associations in the Vitamin D Receptor and Colorectal Cancer in African Americans and Caucasians

**DOI:** 10.1371/journal.pone.0026123

**Published:** 2011-10-27

**Authors:** Sonia S. Kupfer, Jeffrey R. Anderson, Anton E. Ludvik, Stanley Hooker, Andrew Skol, Rick A. Kittles, Temitope O. Keku, Robert S. Sandler, Clara Ruiz-Ponte, Sergi Castellvi-Bel, Antoni Castells, Angel Carracedo, Nathan A. Ellis

**Affiliations:** 1 Section of Gastroenterology, Department of Medicine, University of Chicago Medical Center, Chicago, Illinois, United States of America; 2 Section of Genetic Medicine, Department of Medicine, University of Chicago Medical Center, Chicago, Illinois, United States of America; 3 Department of Medicine, Institute of Human Genetics, University of Illinois at Chicago, Chicago, Illinois, United States of America; 4 Division of Gastroenterology and Hepatology, Department of Medicine, University of North Carolina, Chapel Hill, North Carolina, United States of America; 5 Galician Public Foundation of Genomic Medicine (FPGMX), CIBERER, Genomic Medicine Group, Hospital Clinico, University of Santiago de Compostela, Santiago de Compostela, Galicia, Spain; 6 Department of Gastroenterology, Hospital Clinic, CIBERehd, IDIBAPS, University of Barcelona, Barcelona, Catalonia, Spain; 7 Department of Pediatrics, Institute of Human Genetics, University of Illinois at Chicago, Chicago, Illinois, United States of America; Howard University, United States of America

## Abstract

Low vitamin D levels are associated with an increased incidence of colorectal cancer (CRC) and higher mortality from the disease. In the US, African Americans (AAs) have the highest CRC incidence and mortality and the lowest levels of vitamin D. Single nucleotide polymorphisms (SNPs) in the vitamin D receptor (*VDR*) gene have been previously associated with CRC, but few studies have included AAs. We studied 795 AA CRC cases and 985 AA controls from Chicago and North Carolina as well as 1324 Caucasian cases and 990 Caucasian controls from Chicago and Spain. We genotyped 54 tagSNPs in *VDR* (46586959 to 46521297 Mb) and tested for association adjusting for West African ancestry, age, gender, and multiple testing. Untyped markers were imputed using MACH1.0. We analyzed associations by gender and anatomic location in the whole study group as well as by vitamin D intake in the North Carolina AA group. In the joint analysis, none of the SNPs tested was significantly associated with CRC. For four previously tested restriction fragment length polymorphisms, only one (referred to as *ApaI*), tagged by the SNP rs79628898, had a nominally significant p-value in AAs; none of these polymorphisms were associated with CRC in Caucasians. In the North Carolina AAs, for whom we had vitamin D intake data, we found a significant association between an intronic SNP rs11574041 and vitamin D intake, which is evidence for a *VDR* gene-environment interaction in AAs. In summary, using a systematic tagSNP approach, we have not found evidence for significant associations between *VDR* and CRC in AAs or Caucasians.

## Introduction

Vitamin D has numerous physiological effects, including effects on regulation of calcium homeostasis, immunity, insulin secretion, and blood pressure. Vitamin D_3_ (cholecalciferol) is formed from the precursor steroid 7-dehydrocholesterol (7-DHC), which is concentrated in the plasma membrane of the basal keratinocytes in the skin [1]. Upon stimulation of sunlight (UVB, 280–320 nm), 7-DHC is converted to vitamin D_3_. Vitamin D_3_ is converted in the liver to 25(OH)D_3_ (calcidiol) and in the tissues to 1,25(OH)_2_D_3_ (calcitriol), which is the most active form of the vitamin. Serum 25(OH)D_3_ level, which is the most widely accepted indicator of vitamin D status, is the sum of dietary/supplementary intake and endogenous synthesis. That said, up to 95% of vitamin D is attributable to synthesis in the skin with sunlight exposure, because there are relatively few dietary sources that contain vitamin D [2]. Factors that negatively impact vitamin D status include lack of sun exposure, lack of vitamin D intake, dark skin, aging, and obesity among others.

Garland and Garland [3] first suggested the vitamin D hypothesis so called because they found a correlation between latitude and colorectal cancer (CRC) prevalence. Multiple studies have since shown that vitamin D status can influence the risk of developing CRC. Meta-analyses of case-control studies have shown vitamin D intake and levels of serum 25(OH)D_3_ are associated with CRC [4], [5] and adenomatous colonic polyps [6]. In several incidence cohort and prevention studies, 25(OH)D_3_ supplementation was found to inhibit colon carcinogenesis [7]–[10]. 25(OH)D_3_ inhibits cell proliferation and induces apoptosis of CRC cell lines, and it has similar effects in the colon in animal models and in humans [11]–[13].

Cutaneous melanin absorbs the UVB wavelengths, and it attenuates the synthesis of vitamin D. In Hollis' 1991 study [14], after treatment with UVB, Caucasian subjects generated the highest levels of serum 25(OH)D_3_ whereas African Americans (AAs) generated the lowest levels. As might be expected in comparisons of persons at similar latitudes, AAs have the lowest levels of serum 25(OH)D_3_ of any US population [15]. Consistent with a negative correlation between vitamin D levels and CRC, AAs have the highest CRC incidence and mortality of any US population. Because low serum 25(OH)D_3_ is associated with CRC, vitamin D status could play an important role in CRC risk in the AA population. Vitamin D may also play an important role in CRC mortality in AAs [16].

The 1,25(OH)_2_D_3_ metabolite binds to and activates the vitamin D receptor (VDR), which regulates transcription of numerous downstream genes. Genetic variants in the *VDR* gene have been previously associated with CRC and colonic adenoma risk; however, *VDR* association results have been inconsistent [17]–[43]. Many studies have focused on polymorphisms of convenience (restriction fragment length polymorphisms—RFLPs—and microsatellites), including variants defined by polymorphisms in *TaqI* (rs731236), *BsmI* (rs1544410), and *ApaI* (rs7975232) restriction-enzyme sites and a polymorphic adenine mononucleotide run [21]–[26], [28], [30], [32]–[34], [37]. Some studies focused on possible functional variants, for example, the *FokI* RFLP (rs10735810) and a polymorphism (rs11568820) in the caudal-related homeodomain protein *Cdx-2* binding element in the promoter of *VDR* [22], [23], [25], [27], [28], [31], [32], [35]–[37], [39]–[41]. Previous work has also suggested that haplotypes that include these variants are associated with CRC [38]. Only two published studies have taken a systematic approach to testing single nucleotide polymorphisms (SNPs) that tag most of the common genetic variation in *VDR*. Using a tagSNP approach, Poynter [42] and Egan [43] and their colleagues did not find significant associations between *VDR* SNPs and CRC or colonic adenoma, respectively, in individuals of European descent. Because the vitamin D pathway could play an important role in CRC in AAs, here we have tested whether *VDR* tagSNPs are associated with CRC in both AAs and Caucasians.

## Materials and Methods

### Ethics Statement

All three studies were approved by their respective institutional review boards, and where appropriate, subjects provided written informed consent.

### Cases and Controls

Cases and controls were obtained from the University of Chicago (UC), the University of North Carolina (UNC) and the Spanish CRC consortium EPICOLON (SP). In total, we included DNA from 795 AA CRC cases (404 UC, 391 UNC) and 985 AA controls (568 UC, 417 UNC) as well as 1324 Caucasian cases (399 UC, 925 SP) and 990 Caucasian controls (367 UC, 623 SP). DNA samples from UC cases and controls were prepared from archived surgical specimens as described previously [44]. Additional UC cases were also obtained prospectively from the oncology clinic between 2006–2007 and UC cancer-free controls from the gastroenterology clinic in 2009 (individuals found to have a normal screening colonoscopy) or from the UC Translational Research Initiative (2005–2008).

Samples from UNC were obtained through a large-scale population-based case-control study of colon and rectal cancer, conducted in a 33 county area in central and eastern North Carolina. Cases were drawn at random from all CRC cases reported to the North Carolina Central Cancer Registry. Controls were selected from North Carolina Division of Motor Vehicle lists if under the age of 65, or from a list of Medicare eligible beneficiaries obtained from the Health Care Financing Administration if over the age of 65, based on sampling probabilities within blocks defined by 5-year age group, sex and race, using the technique of randomized recruitment [45]. Vitamin D intake was determined by daily supplement use and/or dietary intake as determined by the Block food frequency questionnaire [46]. The details of this study have been published previously [47].

Spanish samples were obtained through the EPICOLON project, a prospective, multicentre, population-based epidemiology survey studying the incidence and features of familial and sporadic CRC in the Spanish population [48]. Cases were ascertained from 11 hospitals in Spain. All patients with a *de novo* histologically confirmed diagnosis of colorectal adenocarcinoma between November 2006 and December 2007 were selected. Patients in whom CRC developed in the context of familial adenomatous polyposis or inflammatory bowel disease were excluded. Demographic, clinical and tumor-related characteristics of probands, as well as a detailed family history were obtained using a pre-established questionnaire, and registered in a single database. Cases and controls were matched for sex and age (±5 years) and controls were negative for personal and family cancer history. DNA samples were extracted as previously described [49].

Clinical characteristics (Table 1) were compared between cases and controls by race. Two-sided t-tests were used to compare continuous variables including age and ancestry estimates. Pearson chi-square tests of independence were used to compare categorical variables. Because there was significant heterogeneity between the study groups with respect to age and gender, we adjusted for these parameters in the logistic regression models (see below).

**Table 1:** Clinical characteristics of African American and Caucasian study groups.

		African-Americans		Caucasians	
	Study group	Cases	Controls	Cases	Controls
Number of subjects	**Total**	**795**	**985**	**1317**	**978**
	UC	404	568	399	367
	UNC	391	417		
	Spain			918	611
Mean age, years (SD)	**Total**	**64.5 (11.7)** *	**62.3 (13.2)** *	**68.1 (11.5)** *	**65 (12.3)** *
	UC	67.3 (12.7)	60.2 (15.8)	64.6 (13.1)	61.1 (12.7)
	UNC	61.8 (10.0)	65.2 (9.6)		
	Spain			71.6 (9.9)	68.9 (11.8)
Gender (F/M)	**Total**	**423/372** ∧	**570/412** ∧	**513/803** ∧∧	**446/528** ∧∧
	UC	230/172	375/190	170/229	181/183
	UNC	193/200	195/222		
	Spain			343/574	265/345
% West African ancestry (SD)	**Total**	**84.1 (14.0)**	**85.5 (14.3)**		
	UC	85.6 (14.8)	87.8 (13.7)	1.0 (1.5)	0.9 (1.3)
	UNC	82.7 (12.9)	82.4 (14.6)		
Anatomic site, n (%)					
Colon	**Total**	**605 (76.4)**		**814 (68.3)**	
	UC	337 (84.0)		248 (62.3)	
	UNC	268 (68.5)			
	Spain			566 (71.4)	
Rectum	**Total**	**187 (23.6)**		**377 (31.7)**	
	UC	64 (16.0)		150 (37.7)	
	UNC	123 (31.5)			
	Spain			227 (28.6)	

UC, University of Chicago; UNC, University of North Carolina; Spain, Epicolon Spanish consortium; SD, standard deviation; F, female; M, male; colon, proximal to and including sigmoid colon cancers; rectum, rectal and rectosigmoid cancers.

All p-values for heterogeneity between total cases and controls ≥0.05 unless otherwise indicated by footnote symbols.

*p-value for heterogeneity, <0.001;

∧ p-value, 0.04;

∧∧ p-value, 0.001.

### SNP Selection

*VDR* tagSNPs were selected for genotyping from HapMap (NCBI build 36) and NIEHS' Gene SNPs database using Haploview [50]. TagSNPs were determined using Yoruban (YRI) data from chromosome 12 base pairs 46586959 (5′ end) to 46521297 (3′ end) which includes 1.9 kb on the 5′ end to capture the promoter region. The following criteria were used to select tagSNPs: minor allele frequency >5% (in Yoruban population) and pair wise r^2^>0.80. We identified 60 tagSNPs.

### Genotyping

Germline DNA from normal tissue was prepared from both archived formalin-fixed surgical specimens and from blood specimens as described [46], [51]. Of the 60 tagSNPs identified, using the Sequenom MassARRAY platform, we could develop genotyping assays on 54 SNPs. The method used for this platform was described previously [51]. In UC and UNC AA DNA samples, we genotyped 100 ancestry informative markers (AIMs) from which we calculated individual estimates of global West African ancestry [52].

We tested for departures from Hardy-Weinberg equilibrium (HWE) in cases and controls separately. We excluded SNPs with HWE p-values<0.001. We used this cut-off because it is above the significance threshold after adjustment for multiple testing. Additional quality control measures included SNP and individual missingness >10%. In AAs, no SNPs failed the HWE threshold and one SNP failed the SNP missingness threshold. As result, 53 tagSNPs were included for analysis of the AA study group. In the Caucasian study group, all 54 tagSNPs were included in the analysis after quality controls. Genotyping rates were >98.7% for all samples. Concordance rates for 24 duplicate samples were 99.9%.

### Genetic ancestry estimation

Global individual ancestry was determined for each individual in the UC and UNC study groups using 100 AIMs for European and West African ancestry [51]. Individual ancestry estimates were obtained from the genotype data using the Markov Chain Monte Carlo (MCMC) method implemented in the program STRUCTURE 2.1 [53]. STRUCTURE 2.1 assumes an admixture model using prior population information and independent allele frequencies. The MCMC model was run using K = 3 populations (58 Europeans, 67 Native Americans and 62 West Africans) and a burn-in length of 30,000 iterations followed by 70,000 replications.

### Statistical Analysis

We tested *VDR* tagSNPs for association with CRC in the entire case-control series (UC, UNC and Spain) using the pooled analysis methodology described in Zeggini et al. [54]. For this combined analysis, we analyzed SNPs with MAF>5% in both the AA and Caucasian populations (n = 32). We also analyzed associations separately in the combined AA group (UC and UNC) and in the combined Caucasian group (UC and Spain). Associations were further analyzed in the individual study groups from UC (separately by race), UNC and Spain. We calculated odds ratios and 95% confidence intervals using logistic regression assuming a log-additive genetic model. For selected SNPs, we tested dominant and recessive genetic models as well. In the AA study groups (UC and UNC), we controlled for individual admixture by including global West African ancestry estimates in the logistic regression models. In all individuals, we controlled for age and gender in the logistic regression model. We determined the significance of each SNP association empirically using 1000 permutations. These association tests were performed using the program PLINK [55].

We imputed untyped SNPs using MACH v1.0 [56], [57]. MACH uses a MCMC-based algorithm to infer genotypes for individuals. We identified an approximately 2 Mb region on chromosome 12 (45553633 to 47551615 bp) which included *VDR*. Since many of the markers typed in our tagSNP set were missing in the phased haplotypes of the HapMap Data Phase III/Rel#3 (May10), we downloaded the most recent SNP genotype data (HapMap Data Rel#28 Phase II and III) for both the YRI and CEU populations and phased each set in the YRI and CEU separately using MACH and employing parameters -rounds 50 and -states 500. We then imputed genotypes in the African-American study groups using the phased haplotypes from the YRI population and genotypes in the Caucasian study groups using the phased haplotypes from the CEU population. For all two imputations, we used the following parameters: -greedy, -rounds 100, -autoflip. We included only markers with r^2^>0.50 as a quality control and HWE>10^−5^. Imputed genotypes were tested for association as described above.

Sub-group analysis was done by gender and anatomic site (colon versus rectum) in the AA and Caucasian groups separately. For the UNC study group, for which we had supplement and dietary intake data, we tested for SNP associations by vitamin D intake. We defined vitamin D intake as a categorical variable based on a threshold daily intake of >100 IU. To test for SNP-vitamin D intake interaction, we performed a Breslow-Day test for between cluster heterogeneity. We corrected for multiple hypothesis testing using a Bonferroni correction. These association tests were also performed using PLINK [55].

## Results

### Analysis of tagged and imputed SNPs

Under the assumption that common genetic variants in *VDR* influence risk of developing CRC, we performed a combined analysis of the entire study group, including AAs (UC and UNC) and Caucasians (UC and Spain). This analysis did not reveal evidence of association between *VDR* tagSNPs and CRC (Table 2).

**Table 2:** Combined analysis for *VDR* associations in African Americans and Caucasians.

SNP	Bp	RFLP	Combined OR	Z-statistic	Combined p-value
rs739837	46524488		0.97	−0.67	0.50
rs731236	46525024	*TaqI*	1.01	0.17	0.87
rs7962898	46529104	*ApaI*	1.09	1.72	0.08
rs7967152	46530451		0.97	−0.70	0.48
rs2239185	46530826		0.95	−1.05	0.29
rs7971418	46531502		0.92	−1.69	0.09
rs7975128	46532095	*BsmI*	1.07	1.41	0.16
rs7305032	46536127		0.97	−0.67	0.50
rs11168267	46537809		1.06	0.62	0.53
rs11168268	46538079		0.99	−0.11	0.91
rs2248098	46539623		0.92	−1.66	0.10
rs987849	46540943		0.95	−1.03	0.30
rs2239182	46541678		0.99	−0.30	0.76
rs2107301	46541837		1.01	0.22	0.82
rs1540339	46543593		0.96	−0.69	0.49
rs12717991	46545393		0.99	−0.17	0.87
rs2189480	46550095		0.99	−0.26	0.79
rs3819545	46551273		0.97	−0.64	0.52
rs3782905	46552434		1.01	0.27	0.78
rs10735810	46559162	*FokI*	1.00	−0.04	0.97
rs2408876	46559832		0.96	−0.87	0.38
rs2254210	46559981		1.05	0.99	0.32
rs11574044	46562101		1.03	0.46	0.64
rs2238136	46563980		0.95	−0.90	0.37
rs2238135	46564457		0.93	−1.36	0.17
rs2853564	46564754		1.11	1.82	0.07
rs11168287	46571681		0.93	−1.52	0.13
rs4328262	46571915		0.95	−0.96	0.33
rs4334089	46572282		1.02	0.31	0.76
rs3890733	46575640		1.09	1.44	0.15
rs7302235	46579105		0.98	−0.44	0.66
rs7136534	46580893		0.98	−0.33	0.74

SNP, single nucleotide polymorphism; bp, base pair; RFLP, restriction fragment length polymorphism; OR, combined odds ratio.

Combined analysis performed according to Zeggini et al. (ref 54).

**Figure 1:**
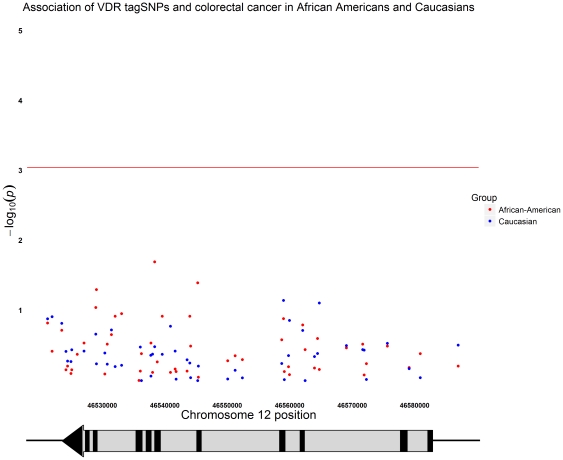
Association of *VDR* tagSNPs in African Americans and Caucasians. Plot of −log_10_ transformed p-values calculated for *VDR* tagSNPs and adjusted for age, gender and ethnic admixture (for the African American study group only) versus nucleotide position on chromosome 12. The arrow depicts the *VDR* gene, which is transcribed in direction from the telomere towards the centromere. The line shows p-value threshold accounting for number of tests (9×10^−4^) based on a Bonferroni correction. Results for African Americans are shown in red and Caucasians in blue.

In AAs, combined analysis (UC and UNC) of *VDR* tagSNPs did not provide evidence for association between *VDR* and CRC. (Figure 1 and Supplementary Table S1). After adjustment for age, gender, and West African ancestry, three SNPs (rs7962898, rs12308082, and rs11574065) had p-values≤0.05, but these p-values were not significant after adjustment for multiple testing. The SNP with the smallest p-value was rs12308082 (OR = 0.74, 95% CI [0.57–0.96]; p = 0.02). Analysis of the individual AA study groups were consistent with the combined AA study group, in as much as each SNP that was nominally significant in the combined study group was nominally significantly associated with CRC in one of the two AA study groups (Supplementary Table S2). No SNP was nominally significant in both study groups. We noted that both rs12308082 and the SNP with the second smallest p-value, rs11574065, (p = 0.04) had relatively low allele frequencies in controls (0.09 and 0.02, respectively); these SNPs were monomorphic in Caucasians.

In the Caucasian study group, combined analysis (UC and SP) of *VDR* tagSNPs again did not provide evidence for association between *VDR* and CRC. (Figure 1 and Supplementary Table S1). After adjustment for age and gender, two SNPs trended toward significance (rs107783218 and rs2853564), but as with the AA analyses, no SNPs were significantly associated with CRC after adjustment for multiple testing. In the UC Caucasian study group, there was one SNP that had a p-value<0.05 (rs10783218, p = 0.02) (Supplementary Table S3), but none of the SNPs had p-values<0.05 in the Spanish Caucasian study group. We note that because the tagSNPs were selected based on Yoruban genotype data, several SNPs were monomorphic or rare in Caucasians.

**Figure 2:**
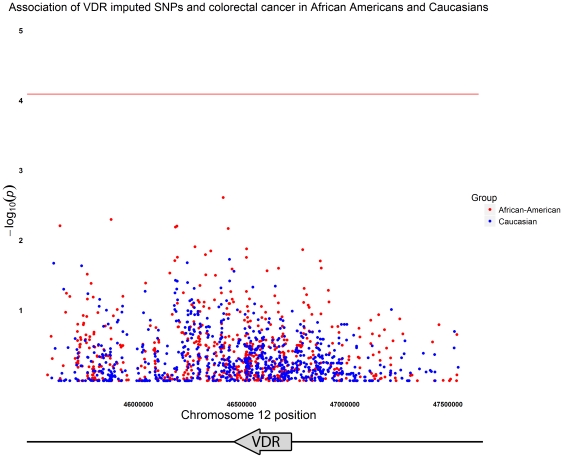
Association of *VDR* imputed SNPs in African Americans and Caucasians. Plot of −log_10_ transformed p-values calculated on imputed genotypes and adjusted for age, gender and ethnic admixture (for the African American study group only) versus nucleotide position on chromosome 12. The line shows p-value threshold accounting for number of tests (8×10^−5^) based on a Bonferroni correction. Results for African Americans are shown in red and Caucasians in blue.

Using tagSNPs, we imputed untyped markers across a 2 Mb region on chromosome 12 including *VDR*. In total, we imputed 2236 SNPs in AAs and 2185 SNPs in Caucasians after applying quality controls. After filtering out monomorphic alleles, we tested 664 imputed SNPs in AAs and 820 imputed SNPs in Caucasians. We did not find evidence for associations between any imputed SNPs and CRC in either study group after adjustment for multiple testing (Figure 2).

### Analysis of previously tested RFLPs

We tested SNPs that were previously studied in RFLP studies—two directly, referred to as *FokI* (rs10735810) and *TaqI* (rs731236), and two indirectly with tagSNPs, referred to as *BsmI* (tagSNP rs7975128, r^2^ = 1) and *ApaI* (tagSNP rs7962898, r^2^ = 0.975). In the combined analysis of the entire study group, the SNP rs7962898 tagging *ApaI* had the smallest combined p-value of 0.08 (Table 2). None of the other previously reported RFLP SNPs (*FokI*, *TaqI* and *BsmI*) showed evidence for association with CRC in this analysis. We next analyzed additive, dominant and recessive models in the AA and Caucasian study groups separately (Table 3). In AAs (Table 3A), the *FokI* and *TaqI* SNPs were not significantly associated with CRC; however, the SNP tagging *ApaI* was nominally significant under the additive model (p = 0.05). In the analysis of AAs by individual institution (UC vs. UNC), the SNP that tagged *BsmI* was nominally associated with CRC in the UC study group (p = 0.05) but not in the UNC study group (p = 0.62) (Supplementary Table S2). None of these SNPs were nominally significant in Caucasians under any genetic model tested (Table 3B).

**Table 3:** *VDR* RFLP associations by genetic model.

SNP	RFLP	Allele	Model	OR [95% CI]	P-value
**(A) African Americans**					
rs731236	*TaqI*	C	Additive	0.98 [0.84–1.14]	0.79
			Dominant	1.03 [0.85–1.26]	0.74
			Recessive	0.81 [0.56–1.15]	0.24
rs7962898	*ApaI*	T	Additive	1.15 [1.00–1.33]	0.05
			Dominant	1.19 [0.97–1.46]	0.09
			Recessive	1.24 [0.93–1.65]	0.14
rs7975128	*BsmI*	T	Additive	1.13 [0.97–1.31]	0.12
			Dominant	1.16 [0.95–1.41]	0.15
			Recessive	1.19 [0.85–1.68]	0.31
rs10735810	*FokI*	T	Additive	0.97 [0.82–1.15]	0.74
			Dominant	1.01 [0.82–1.23]	0.94
			Recessive	0.76 [0.47–1.24]	0.27
**(B) Caucasians**					
rs731236	*TaqI*	C	Additive	1.04 [0.91–1.19]	0.53
			Dominant	1.13 [0.93–1.36]	0.21
			Recessive	0.94 [0.73–1.21]	0.64
rs7962898	*ApaI*	C	Additive	0.96 [0.85–1.10]	0.57
			Dominant	0.94 [0.77–1.16]	0.58
			Recessive	0.96 [0.77–1.19]	0.72
rs7975128	*BsmI*	T	Additive	1.03 [0.91–1.18]	0.63
			Dominant	1.14 [0.94–1.37]	0.18
			Recessive	0.89 [0.70–1.15]	0.38
rs10735810	*FokI*	T	Additive	1.00 [0.87–1.14]	0.96
			Dominant	0.95 [0.79–1.14]	0.56
			Recessive	1.10 [0.85–1.44]	0.47

SNP, single nucleotide polymorphism; RFLP, restriction fragment length polymorphism; OR, odds ratio; CI, confidence interval.

### Sub-group analyses

There have been previous reports of associations when stratified by gender [18]; consequently, we further analyzed our association results by gender in the AA and Caucasians study groups. Among AA females, there was one SNP rs11168264 that was nominally significant (p = 0.05). This SNP is rare in Caucasians. No SNPs were noted to be associated in the Caucasian female group (Supplementary Figure S1 & Supplementary Table S4). In males, there were several associations noted. Among AA males, rs11574065 (p = 0.02) and rs11574050 (p = 0.009) showed evidence of association. Among Caucasian males, rs2254210 (p = 0.005) and rs2853564 (p = 0.03) showed evidence for association with CRC. However, none of these p-values were significant after adjustment for multiple testing.

Because previous reports have suggested associations with anatomic sites in the colon [37], we next considered associations by anatomic site in the large bowel (Supplementary Figure S2 & Supplementary Tables S5 and S6). Our sub-group analysis showed associations primarily in the AA rectal cancer group. In the combined AA group, four SNPs had adjusted p-values less than 0.05 (Supplementary Table S5). Of these, two SNPs rs12314197 and rs7962898 are tagSNPs for *ApaI*. Considering rectal cancer associations by center (Supplementary Table S6), we found that the *ApaI* tagSNPs were associated with rectal cancer in the UNC study group; while, the other two SNPs rs3890733 and rs7302235 were associated in the UC study group. However, none of these SNPs were significant after adjustment for multiple testing. In the Caucasian study group, one SNP rs2853564 had evidence of association with colon cancer (p = 0.01); while not significant, this association showed the smallest p-value after taking multiple testing into account (p = 0.28).

For the UNC study group, we tested for differences in SNP associations by vitamin D intake. We found a significant association for an intronic SNP rs11574041 and vitamin D intake (Table 4). In particular, there was significant OR heterogeneity between individuals with vitamin D intake compared to those with no meaningful intake (OR 0.30 vs. 1.05 respectively, Breslow-Day p = 0.004). We noted a protective effect of the A allele in individuals with high vitamin D intake (f_A_ = 0.15 vs. f_U_ = 0.05, p = 9×10^−4^) which was significant when accounting for the number of SNPs tested by Bonferroni correction (p = 9.3×10^−4^).

**Table 4:** Association of rs11574041 A allele with CRC by vitamin D intake in UNC study group.

Vitamin D intake	No. cases	No. controls	Freq. cases	Freq. controls	OR	P-value
≥100 IU	232	214	0.05	0.15	0.30	0.0009
<100 IU	506	582	0.09	0.09	1.05	0.84

No., number; Freq., allele frequency; OR, odds ratio.

## Discussion

Study of *VDR* genetic variants for association with CRC in the AA population is important given the overall higher CRC incidence and mortality as well as lower vitamin D levels in this population compared to other US populations. In our association study using tagSNPs and imputed SNPs in a large group of AAs and Caucasians, we did not find evidence of significant associations between CRC and common genetic variants in *VDR*. Our results were similar in both the combined analysis and the analysis of ethnically-related and individual study groups. Moreover, in our results, we found no significant associations in our sub-group analysis by gender or anatomic site. Overall, our results are in agreement with two recent reports in Caucasians that also reported no evidence for associations between *VDR* tagSNPs and CRC [42] or colonic adenomas [43].

Because previous studies had sometimes found associations between CRC and VDR RFLPs [21]–[28], [30]–[37], [39]–[41], we performed additional genetic analyses on some of these variants; however, *FokI* (rs10735810), *TaqI* (rs731236), and *BsmI* (rs1544410) were not significantly associated with CRC in any study group. In our study, we found a nominally significant association in AA CRC with rs7962898, a SNP that tags the RFLP *ApaI*. Previous studies have found an association with *ApaI* and CRC in non-AA populations, though sample sizes were small and both significant and non-significant results have been reported [21], [26]. Given the lack of evidence for association between CRC and rs7962898 in Caucasians in the present study, we interpret our nominally significant p-value with rs7962898 in AAs cautiously. The association has not been reported previously in this population, and it was observed in only one of the two study groups that we analyzed (UC but not UNC).

In our UNC study group, we note a significant association between an intronic SNP rs11574041 and vitamin D intake. To our knowledge, this association has not been previously reported. While this result suggests a gene-environment interaction, it should be interpreted with caution due to the small sample size and limited data of supplemental and dietary intake available for this analysis. In previous case-control studies, vitamin D intake (both dietary and supplemental) has not been an adequate proxy for vitamin D serum levels and, therefore, probably does not reflect vitamin D status. Our results require further study in a larger sample preferably with data of vitamin D serum levels.

In the present study, there are several possible explanations for the overall lack of significant associations. SNPs in the *VDR* gene on their own may not increase CRC risk; however, if SNPs are playing a role, they may interact with other SNPs or with environmental factors to increase risk. Some differences in associations between African American populations from different geographic locations (UC vs. UNC) were detected, and these differences potentially could be explained by environmental differences between the two groups. Given that control of serum 25(OH)D_3_ levels have both genetic and environmental determinants, we hypothesize that there are important gene-environment interactions that together increase risk of CRC. In the present study, we did not have access to environmental measures of vitamin D intake for the UC and Spanish study groups, nor did we have serum 25(OH)D_3_ levels for any of the study groups.

Alternatively, rare variants in *VDR* may increase CRC risk. In such a genetic model, multiple rare variants with stronger effects (e.g., ORs>1.5) could increase individual risk of CRC such that earlier and more frequent colonoscopic screening would be recommended. Rare variants are not well assessed using a tagSNP approach, and, in general, larger association studies or family-based studies are required to identify associations with rare variants. We note that the two variants in *VDR* with the smallest p-values in the present study were relatively rare.

The strengths of the current study are a large sample size including AAs as well as a comprehensive tagSNP approach to capture nearly all common variation in the *VDR* gene. Moreover, we used imputation to obtain genotypes for untyped SNPs, thereby increasing coverage of the *VDR* gene and surrounding regions on chromosome 12. Weaknesses of this study include lack of measures of serum vitamin D levels in all subjects; such data would potentially uncover interactions between genotype and environment. Moreover, we did not include SNPs that tag the Cdx2 polymorphism in the initial tagSNP analysis, which has yielded positive results in CRC in a previous study [17]. However, our imputation analysis included the region harboring the Cdx2 polymorphism and did not show significant associations. Future studies should include such environmental data such as vitamin D intake and serum levels to determine if gene-environment interactions influence CRC. In addition, there have been reports of novel candidate regions associated with vitamin D levels from genome-wide association and ChIP-sequencing studies that should be tested for genetic association in CRC [58], [59].

In summary, we found no compelling evidence for associations between CRC and genetic polymorphisms in *VDR*. Although we observed a nominally significant CRC association with a SNP that is highly correlated with *ApaI* in AAs, this association was observed in only one of the two AA study groups; moreover, associations were not detected between CRC and other *VDR* tagSNPs in AAs. In Caucasians, associations were not detected between CRC and either previously reported *VDR* RFLPs or *VDR* tagSNPs. A possible association between vitamin D intake and rs11574041 in AA CRC requires further investigation.

## Supporting Information

Figure S1 **Association of** ***VDR*** **tagSNPs in African Americans and Caucasians by gender: (A) females and (B) males.** Plot of −log_10_ transformed p-values calculated for *VDR* tagSNPs and adjusted for age and ethnic admixture (for the African American study group only) versus nucleotide position on chromosome 12. The arrow depicts the *VDR* gene, which is transcribed in direction from the telomere towards the centromere. The line shows p-value threshold accounting for number of tests (9×10^−4^) based on a Bonferroni correction. Results for African Americans are shown in red and Caucasians in blue.
(TIF)

Figure S2 **Association of** ***VDR*** **tagSNPs in African Americans and Caucasians by anatomic site: (A) colon cancer and (B) rectal cancer.** Plot of −log_10_ transformed p-values calculated for *VDR* tagSNPs and adjusted for age, gender and ethnic admixture (for the African American study group only) versus nucleotide position on chromosome 12. The arrow depicts the *VDR* gene, which is transcribed from right to left on the chromosome. The line shows p-value threshold accounting for number of tests (9×10^−4^) based on a Bonferroni correction. Results for African Americans are shown in red and Caucasians in blue.
(TIF)

Table S1 ***VDR*** **associations by ancestry.**
(DOCX)

Table S2 ***VDR*** **associations in African Americans by center.**
(DOCX)

Table S3 ***VDR*** **associations in Caucasians by center.**
(DOCX)

Table S4 ***VDR*** **associations by gender: (A) females and (B) males.**
(DOCX)

Table S5 ***VDR*** **associations by anatomic site: (A) colon cancer (B) rectal cancer.**
(DOCX)

Table S6 ***VDR*** **associations in African Americans by anatomic site and center.**
(DOCX)
