# Supplementary figures and images for: Genetic Associations in the Vitamin D Receptor and Colorectal Cancer in African Americans and Caucasians
Source: PLoS One. 2011 Oct 27;6(10):e26123. doi: 10.1371/journal.pone.0026123 (PMC3203108; doi:10.1371/journal.pone.0026123)

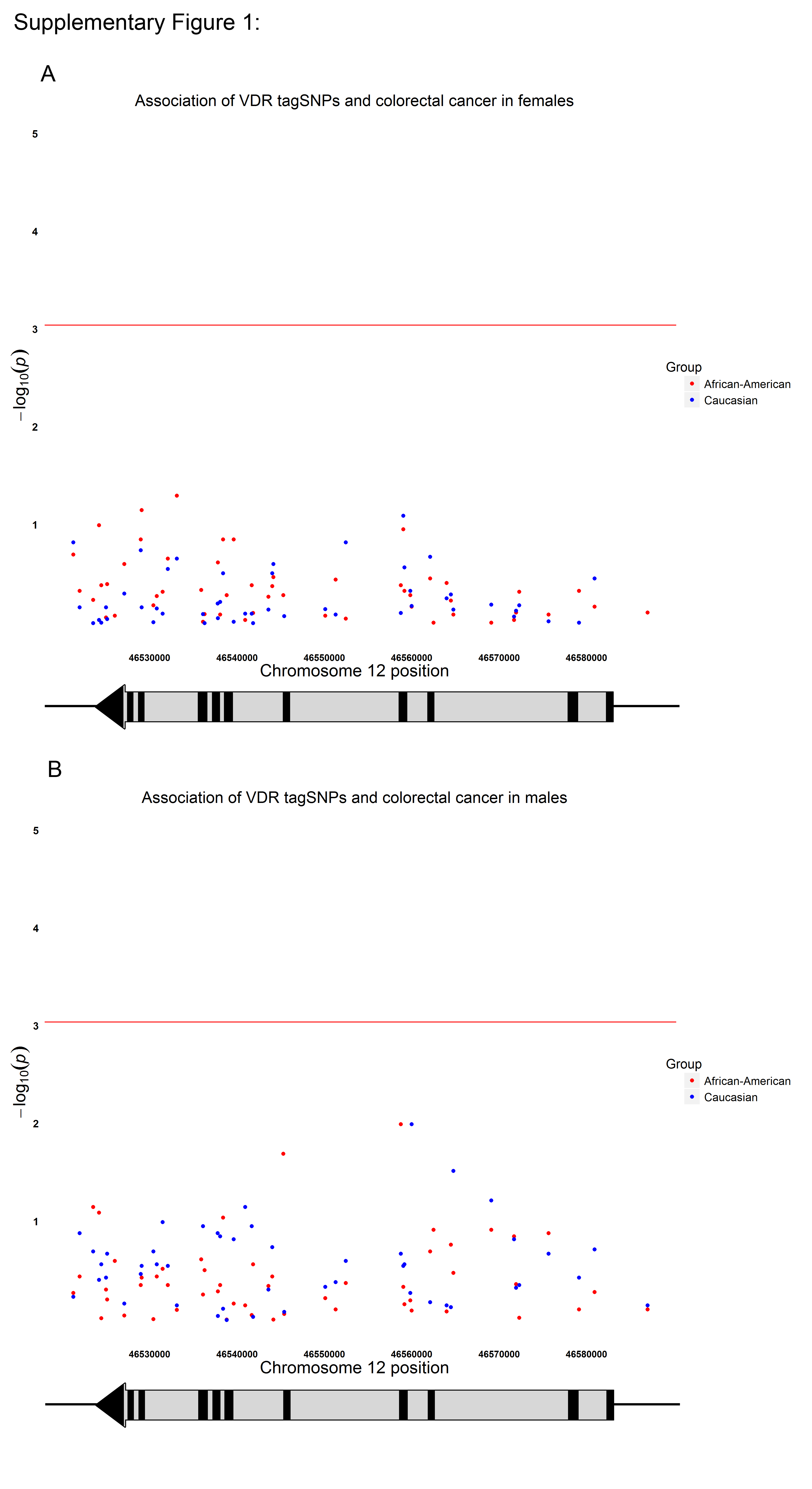

Supplement: Figure S1 — Association of VDR tagSNPs in African Americans and Caucasians by gender: (A) females and (B) males. Plot of −log10 transformed p-values calculated for VDR tagSNPs and adjusted for age and ethnic admixture (for the African American study group only) versus nucleotide position on chromosome 12. The arrow depicts the VDR gene, which is transcribed in direction from the telomere towards the centromere. The line shows p-value threshold accounting for number of tests (9×10^−4^) based on a Bonferroni correction. Results for African Americans are shown in red and Caucasians in blue. (TIF) [file pone.0026123.s001.tif]

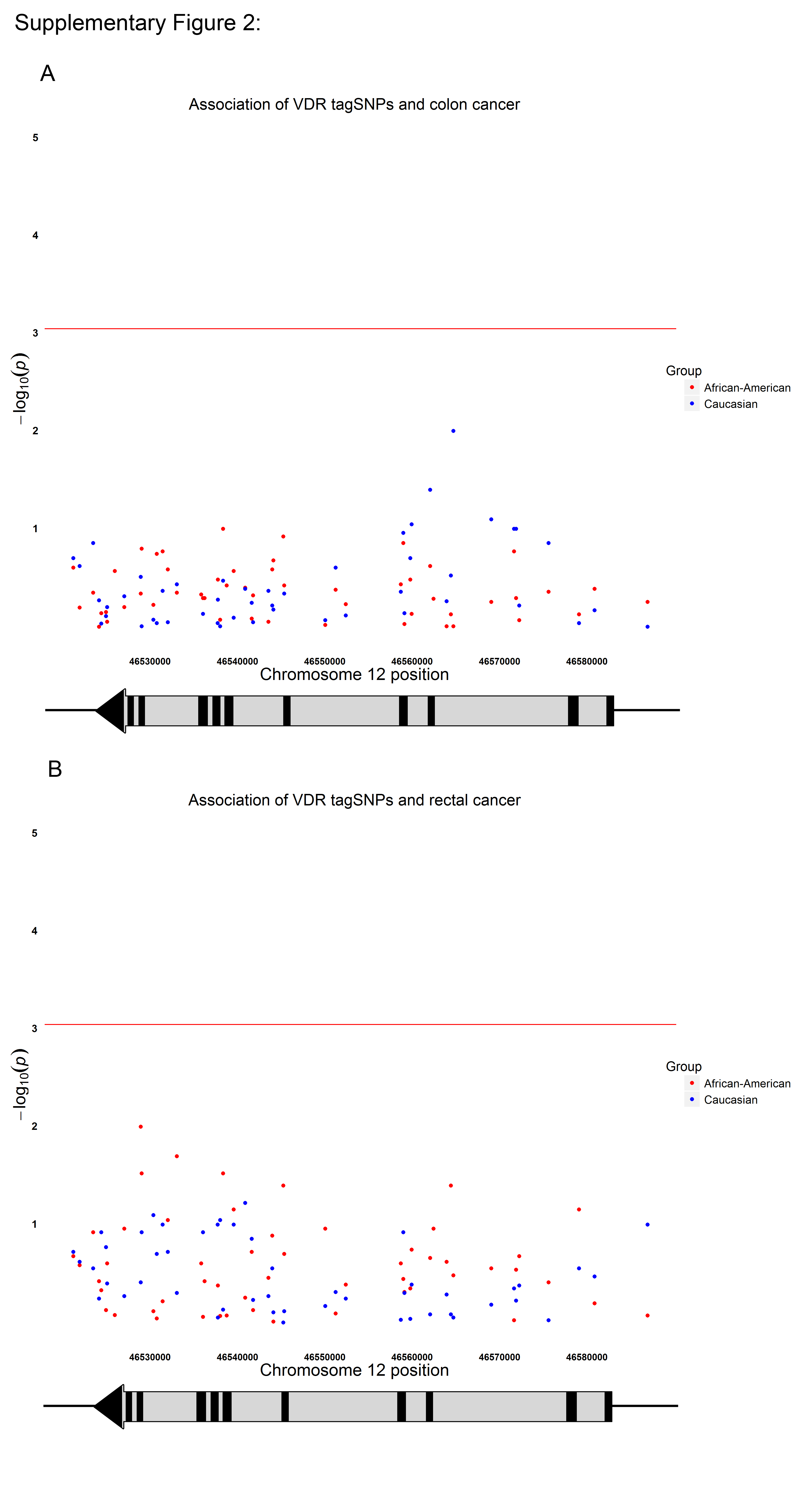

Supplement: Figure S2 — Association of VDR tagSNPs in African Americans and Caucasians by anatomic site: (A) colon cancer and (B) rectal cancer. Plot of −log10 transformed p-values calculated for VDR tagSNPs and adjusted for age, gender and ethnic admixture (for the African American study group only) versus nucleotide position on chromosome 12. The arrow depicts the VDR gene, which is transcribed from right to left on the chromosome. The line shows p-value threshold accounting for number of tests (9×10^−4^) based on a Bonferroni correction. Results for African Americans are shown in red and Caucasians in blue. (TIF) [file pone.0026123.s002.tif]
