# Supplementary material for: Genetic Associations in the Vitamin D Receptor and Colorectal Cancer in African Americans and Caucasians
Source: PLoS One. 2011 Oct 27;6(10):e26123. doi: 10.1371/journal.pone.0026123 (PMC3203108; doi:10.1371/journal.pone.0026123)
Supplement: Table S3 — VDR associations in Caucasians by center. (DOCX) [file pone.0026123.s005.docx]

| **Supplementary Table S3: *VDR* associations in Caucasians by center.** | | | | | | | | | | | |
| --- | --- | --- | --- | --- | --- | --- | --- | --- | --- | --- | --- |
| Center | SNP | RFLP | Allele | Freq. cases | Freq. controls | Inds. | OR* | L95 | U95 | P-value* | Permuted p-value |
| UC | rs11574141 |  | C | 0.00 | 0.00 | 706 | 2.88 | 0.29 | 28.29 | 0.36 | 1.00 |
| Spain | rs11574141 |  | C | 0.01 | 0.00 | 1300 | 2.32 | 0.54 | 10.04 | 0.26 | 1.00 |
| UC | rs2853563 |  | A | 0.03 | 0.03 | 699 | 0.91 | 0.50 | 1.68 | 0.76 | 1.00 |
| Spain | rs2853563 |  | A | 0.09 | 0.08 | 1270 | 1.20 | 0.88 | 1.62 | 0.24 | 1.00 |
| UC | rs7954412 |  | G | 0.00 | 0.00 | NA | NA | NA | NA | NA | NA |
| Spain | rs7954412 |  | G | 0.00 | 0.00 | NA | NA | NA | NA | NA | NA |
| UC | rs3858733 |  | C | 0.03 | 0.03 | 706 | 0.84 | 0.46 | 1.54 | 0.57 | 1.00 |
| Spain | rs3858733 |  | C | 0.08 | 0.08 | 1294 | 1.09 | 0.80 | 1.47 | 0.60 | 1.00 |
| UC | rs739837 |  | C | 0.46 | 0.49 | 706 | 0.93 | 0.75 | 1.14 | 0.48 | 1.00 |
| Spain | rs739837 |  | C | 0.46 | 0.46 | 1292 | 0.99 | 0.84 | 1.16 | 0.86 | 1.00 |
| UC | rs731236 | *TaqI* | C | 0.39 | 0.37 | 704 | 1.04 | 0.84 | 1.28 | 0.75 | 1.00 |
| Spain | rs731236 | *TaqI* | C | 0.39 | 0.38 | 1270 | 1.04 | 0.88 | 1.23 | 0.61 | 1.00 |
| UC | rs11574114 |  | A | 0.03 | 0.03 | 706 | 0.88 | 0.48 | 1.61 | 0.69 | 1.00 |
| Spain | rs11574114 |  | A | 0.08 | 0.08 | 1289 | 1.08 | 0.80 | 1.46 | 0.61 | 1.00 |
| UC | rs11574110 |  | A | 0.00 | 0.00 | NA | NA | NA | NA | NA | NA |
| Spain | rs11574110 |  | A | 0.00 | 0.00 | NA | NA | NA | NA | NA | NA |
| UC | rs11574105 |  | A | 0.00 | 0.00 | NA | NA | NA | NA | NA | NA |
| Spain | rs11574105 |  | A | 0.00 | 0.00 | NA | NA | NA | NA | NA | NA |
| UC | rs12314197 |  | C | 0.00 | 0.00 | 706 | 2.88 | 0.29 | 28.29 | 0.36 | 1.00 |
| Spain | rs12314197 |  | C | 0.01 | 0.01 | 1298 | 1.44 | 0.53 | 3.91 | 0.47 | 1.00 |
| UC | rs7962898 | *ApaI* | C | 0.47 | 0.49 | 696 | 0.92 | 0.75 | 1.14 | 0.45 | 1.00 |
| Spain | rs7962898 | *ApaI* | C | 0.47 | 0.47 | 1259 | 0.99 | 0.84 | 1.17 | 0.92 | 1.00 |
| UC | rs7967152 |  | A | 0.46 | 0.49 | 702 | 0.92 | 0.74 | 1.13 | 0.42 | 1.00 |
| Spain | rs7967152 |  | A | 0.46 | 0.46 | 1291 | 0.97 | 0.82 | 1.14 | 0.69 | 1.00 |
| UC | rs2239185 |  | C | 0.46 | 0.49 | 703 | 0.92 | 0.75 | 1.14 | 0.44 | 1.00 |
| Spain | rs2239185 |  | C | 0.46 | 0.46 | 1273 | 1.00 | 0.85 | 1.18 | 0.97 | 1.00 |
| UC | rs7971418 |  | C | 0.46 | 0.49 | 703 | 0.91 | 0.74 | 1.13 | 0.40 | 1.00 |
| Spain | rs7971418 |  | C | 0.44 | 0.46 | 1252 | 0.93 | 0.78 | 1.10 | 0.38 | 1.00 |
| UC | rs7975128 | *BsmI* | T | 0.40 | 0.38 | 705 | 1.06 | 0.86 | 1.31 | 0.61 | 1.00 |
| Spain | rs7975128 | *BsmI* | T | 0.39 | 0.39 | 1299 | 1.02 | 0.86 | 1.21 | 0.80 | 1.00 |
| UC | rs11168264 |  | C | 0.00 | 0.00 | NA | NA | NA | NA | NA | NA |
| Spain | rs11168264 |  | C | 0.01 | 0.01 | NA | NA | NA | NA | NA | NA |
| UC | rs7966569 |  | C | 0.00 | 0.00 | NA | NA | NA | NA | NA | NA |
| Spain | rs7966569 |  | C | 0.00 | 0.00 | NA | NA | NA | NA | NA | NA |
| UC | rs7305032 |  | C | 0.42 | 0.45 | 704 | 0.91 | 0.74 | 1.12 | 0.37 | 1.00 |
| Spain | rs7305032 |  | C | 0.39 | 0.40 | 1293 | 0.98 | 0.83 | 1.16 | 0.83 | 1.00 |
| UC | rs11574087 |  | T | 0.00 | 0.00 | NA | NA | NA | NA | NA | NA |
| Spain | rs11574087 |  | T | 0.00 | 0.00 | NA | NA | NA | NA | NA | NA |
| UC | rs11168266 |  | G | 0.43 | 0.46 | 690 | 0.92 | 0.75 | 1.14 | 0.44 | 1.00 |
| Spain | rs11168266 |  | G | 0.41 | 0.41 | 1295 | 0.99 | 0.84 | 1.17 | 0.92 | 1.00 |
| UC | rs11168267 |  | T | 0.10 | 0.09 | 706 | 1.08 | 0.75 | 1.56 | 0.68 | 1.00 |
| Spain | rs11168267 |  | T | 0.09 | 0.10 | 1298 | 0.92 | 0.70 | 1.21 | 0.56 | 1.00 |
| UC | rs11168268 |  | C | 0.43 | 0.46 | 704 | 0.92 | 0.74 | 1.13 | 0.40 | 1.00 |
| Spain | rs11168268 |  | C | 0.41 | 0.41 | 1251 | 1.00 | 0.85 | 1.18 | 0.97 | 1.00 |
| UC | rs12308082 |  | T | 0.00 | 0.00 | NA | NA | NA | NA | NA | NA |
| Spain | rs12308082 |  | T | 0.00 | 0.00 | NA | NA | NA | NA | NA | NA |
| UC | rs2853560 |  | T | 0.00 | 0.00 | NA | NA | NA | NA | NA | NA |
| Spain | rs2853560 |  | T | 0.00 | 0.00 | NA | NA | NA | NA | NA | NA |
| UC | rs2248098 |  | T | 0.48 | 0.51 | 703 | 0.93 | 0.75 | 1.14 | 0.46 | 1.00 |
| Spain | rs2248098 |  | T | 0.49 | 0.50 | 1298 | 0.96 | 0.82 | 1.13 | 0.64 | 1.00 |
| UC | rs987849 |  | C | 0.46 | 0.48 | 696 | 0.94 | 0.77 | 1.15 | 0.55 | 1.00 |
| Spain | rs987849 |  | C | 0.43 | 0.45 | 1238 | 0.92 | 0.78 | 1.08 | 0.30 | 1.00 |
| UC | rs2239182 |  | G | 0.50 | 0.46 | 704 | 1.15 | 0.94 | 1.41 | 0.18 | 0.96 |
| Spain | rs2239182 |  | G | 0.49 | 0.50 | 1276 | 1.00 | 0.85 | 1.18 | 1.00 | 1.00 |
| UC | rs2107301 |  | T | 0.29 | 0.29 | 704 | 0.97 | 0.77 | 1.23 | 0.80 | 1.00 |
| Spain | rs2107301 |  | T | 0.30 | 0.29 | 1268 | 1.01 | 0.85 | 1.20 | 0.91 | 1.00 |
| UC | rs1540339 |  | A | 0.38 | 0.39 | 704 | 0.92 | 0.74 | 1.14 | 0.45 | 1.00 |
| Spain | rs1540339 |  | A | 0.38 | 0.37 | 1292 | 0.99 | 0.83 | 1.17 | 0.87 | 1.00 |
| UC | rs2239179 |  | G | 0.42 | 0.41 | 689 | 1.07 | 0.87 | 1.32 | 0.53 | 1.00 |
| Spain | rs2239179 |  | G | 0.42 | 0.43 | 1219 | 1.01 | 0.86 | 1.20 | 0.90 | 1.00 |
| UC | rs11574070 |  | T | 0.00 | 0.00 | NA | NA | NA | NA | NA | NA |
| Spain | rs11574070 |  | T | 0.00 | 0.00 | NA | NA | NA | NA | NA | NA |
| UC | rs11574065 |  | T | 0.00 | 0.00 | NA | NA | NA | NA | NA | NA |
| Spain | rs11574065 |  | T | 0.00 | 0.00 | NA | NA | NA | NA | NA | NA |
| UC | rs12717991 |  | A | 0.38 | 0.40 | 706 | 0.92 | 0.73 | 1.14 | 0.43 | 1.00 |
| Spain | rs12717991 |  | A | 0.38 | 0.37 | 1290 | 1.00 | 0.85 | 1.19 | 0.97 | 1.00 |
| UC | rs2189480 |  | A | 0.37 | 0.35 | 687 | 1.06 | 0.85 | 1.33 | 0.61 | 1.00 |
| Spain | rs2189480 |  | A | 0.36 | 0.35 | 1291 | 0.99 | 0.83 | 1.16 | 0.86 | 1.00 |
| UC | rs3819545 |  | C | 0.39 | 0.40 | 702 | 0.96 | 0.77 | 1.19 | 0.70 | 1.00 |
| Spain | rs3819545 |  | C | 0.40 | 0.39 | 1283 | 0.99 | 0.84 | 1.17 | 0.93 | 1.00 |
| UC | rs3782905 |  | G | 0.33 | 0.33 | 705 | 0.98 | 0.79 | 1.23 | 0.89 | 1.00 |
| Spain | rs3782905 |  | G | 0.31 | 0.32 | 1294 | 1.01 | 0.85 | 1.20 | 0.95 | 1.00 |
| UC | rs11574050 |  | T | 0.05 | 0.04 | 706 | 1.27 | 0.76 | 2.12 | 0.36 | 1.00 |
| Spain | rs11574050 |  | T | 0.06 | 0.06 | 1299 | 0.99 | 0.70 | 1.38 | 0.93 | 1.00 |
| UC | rs10783218 |  | T | 0.05 | 0.02 | 705 | 2.15 | 1.15 | 4.01 | 0.02 | 0.28 |
| Spain | rs10783218 |  | T | 0.03 | 0.03 | 1287 | 1.11 | 0.67 | 1.83 | 0.69 | 1.00 |
| UC | rs10735810 | *FokI* | T | 0.40 | 0.39 | 704 | 1.01 | 0.81 | 1.25 | 0.94 | 1.00 |
| Spain | rs10735810 | *FokI* | T | 0.36 | 0.36 | 1291 | 1.01 | 0.85 | 1.20 | 0.92 | 1.00 |
| UC | rs2408876 |  | G | 0.41 | 0.42 | 700 | 0.96 | 0.77 | 1.19 | 0.70 | 1.00 |
| Spain | rs2408876 |  | G | 0.42 | 0.45 | 1219 | 0.93 | 0.78 | 1.10 | 0.38 | 1.00 |
| UC | rs2254210 |  | T | 0.37 | 0.32 | 703 | 1.25 | 0.99 | 1.58 | 0.06 | 0.65 |
| Spain | rs2254210 |  | T | 0.33 | 0.32 | 1297 | 1.05 | 0.89 | 1.25 | 0.56 | 1.00 |
| UC | rs11574044 |  | G | 0.18 | 0.17 | 687 | 1.13 | 0.84 | 1.52 | 0.42 | 1.00 |
| Spain | rs11574044 |  | G | 0.14 | 0.18 | 1300 | 0.80 | 0.64 | 1.00 | 0.05 | 0.65 |
| UC | rs11574041 |  | A | 0.00 | 0.00 | NA | NA | NA | NA | NA | NA |
| Spain | rs11574041 |  | A | 0.00 | 0.00 | NA | NA | NA | NA | NA | NA |
| UC | rs2238136 |  | A | 0.26 | 0.29 | 704 | 0.86 | 0.68 | 1.09 | 0.21 | 0.98 |
| Spain | rs2238136 |  | A | 0.27 | 0.27 | 1290 | 1.01 | 0.84 | 1.21 | 0.92 | 1.00 |
| UC | rs2238135 |  | C | 0.25 | 0.29 | 702 | 0.83 | 0.66 | 1.06 | 0.14 | 0.93 |
| Spain | rs2238135 |  | C | 0.26 | 0.26 | 1292 | 1.02 | 0.85 | 1.23 | 0.84 | 1.00 |
| UC | rs2853564 |  | C | 0.41 | 0.36 | 705 | 1.23 | 0.99 | 1.53 | 0.06 | 0.69 |
| Spain | rs2853564 |  | C | 0.37 | 0.35 | 1297 | 1.09 | 0.92 | 1.30 | 0.32 | 1.00 |
| UC | rs2853559 |  | T | 0.40 | 0.36 | 696 | 1.19 | 0.96 | 1.48 | 0.12 | 0.90 |
| UC | rs11168287 |  | T | 0.52 | 0.47 | 699 | 1.20 | 0.97 | 1.48 | 0.09 | 0.81 |
| Spain | rs11168287 |  | T | 0.47 | 0.47 | 1285 | 1.00 | 0.85 | 1.18 | 0.99 | 1.00 |
| UC | rs4328262 |  | C | 0.41 | 0.44 | 706 | 0.89 | 0.72 | 1.11 | 0.32 | 1.00 |
| Spain | rs4328262 |  | C | 0.44 | 0.45 | 1276 | 0.96 | 0.81 | 1.13 | 0.59 | 1.00 |
| UC | rs4334089 |  | T | 0.25 | 0.26 | 704 | 0.98 | 0.76 | 1.25 | 0.86 | 1.00 |
| Spain | rs4334089 |  | T | 0.29 | 0.29 | 1289 | 1.00 | 0.84 | 1.19 | 0.97 | 1.00 |
| UC | rs3890733 |  | A | 0.32 | 0.31 | 667 | 1.04 | 0.82 | 1.32 | 0.74 | 1.00 |
| Spain | rs3890733 |  | A | 0.30 | 0.27 | 1241 | 1.13 | 0.94 | 1.36 | 0.20 | 0.99 |
| UC | rs7302235 |  | G | 0.27 | 0.26 | 696 | 1.02 | 0.80 | 1.30 | 0.86 | 1.00 |
| Spain | rs7302235 |  | G | 0.28 | 0.29 | 1210 | 0.94 | 0.79 | 1.12 | 0.46 | 1.00 |
| UC | rs7136534 |  | A | 0.25 | 0.26 | 705 | 0.96 | 0.75 | 1.22 | 0.73 | 1.00 |
| Spain | rs7136534 |  | A | 0.27 | 0.26 | 1288 | 1.00 | 0.84 | 1.20 | 0.98 | 1.00 |
| UC | rs11574002 |  | G | 0.00 | 0.00 | NA | NA | NA | NA | NA | NA |
| Spain | rs11574002 |  | G | 0.00 | 0.00 | NA | NA | NA | NA | NA | NA |
| UC, University of Chicago; Spain, Epicolon consortium; SNP, single nucleotide polymorphism; RFLP, restriction fragment length polymorphism; Freq., allele frequency; Inds., number of individuals included for each analysis; L95, lower boundary 95% confidence interval; U95, upper boundary 95% confidence interval; NA, not applicable due to monomorphic allele.  *OR and p-value adjusted for age, gender and West African ancestry (ancestry only adjusted for in AA group). | | | | | | | | | | | |
