# Supplementary material for: Genetic Associations in the Vitamin D Receptor and Colorectal Cancer in African Americans and Caucasians
Source: PLoS One. 2011 Oct 27;6(10):e26123. doi: 10.1371/journal.pone.0026123 (PMC3203108; doi:10.1371/journal.pone.0026123)
Supplement: Table S4 — VDR associations by gender: (A) females and (B) males. (DOCX) [file pone.0026123.s006.docx]

| **Supplementary Table S4: *VDR* associations by gender: (A) females and (B) males.** | | | | | | | | | | | |
| --- | --- | --- | --- | --- | --- | --- | --- | --- | --- | --- | --- |
| Ancestry | SNP | RFLP | Allele | Freq. cases | Freq. controls | Inds. | OR* | L95 | U95 | P-value* | Permuted p-value |
| **(A) Females** |  |  |  |  |  |  |  |  |  |  |  |
| AA | rs11574141 |  | C | 0.07 | 0.08 | 971 | 0.80 | 0.56 | 1.13 | 0.20 | 1.00 |
| Caucasian | rs11574141 |  | C | 0.01 | 0.00 | 916 | 2.98 | 0.68 | 13.16 | 0.15 | 0.98 |
| AA | rs2853563 |  | A | 0.14 | 0.16 | 964 | 0.91 | 0.70 | 1.17 | 0.47 | 1.00 |
| Caucasian | rs2853563 |  | A | 0.07 | 0.06 | 909 | 1.08 | 0.75 | 1.54 | 0.69 | 1.00 |
| AA | rs7954412 |  | G | 0.05 | 0.06 | 970 | 0.89 | 0.60 | 1.33 | 0.58 | 1.00 |
| Caucasian | rs7954412 |  | G | 0.00 | 0.00 | 926 | NA | NA | NA | 1.00 | 1.00 |
| AA | rs3858733 |  | C | 0.01 | 0.01 | 971 | 0.43 | 0.15 | 1.18 | 0.10 | 0.97 |
| Caucasian | rs3858733 |  | C | 0.07 | 0.07 | 907 | 0.98 | 0.68 | 1.42 | 0.93 | 1.00 |
| AA | rs739837 |  | C | 0.40 | 0.43 | 969 | 0.93 | 0.77 | 1.11 | 0.41 | 1.00 |
| Caucasian | rs739837 |  | C | 0.47 | 0.47 | 906 | 1.00 | 0.83 | 1.20 | 0.99 | 1.00 |
| AA | rs731236 | *TaqI* | C | 0.31 | 0.30 | 967 | 1.02 | 0.83 | 1.25 | 0.88 | 1.00 |
| Caucasian | rs731236 | *TaqI* | C | 0.38 | 0.38 | 884 | 0.96 | 0.79 | 1.17 | 0.69 | 1.00 |
| AA | rs11574114 |  | A | 0.14 | 0.16 | 970 | 0.90 | 0.70 | 1.16 | 0.40 | 1.00 |
| Caucasian | rs11574114 |  | A | 0.07 | 0.07 | 903 | 0.98 | 0.68 | 1.41 | 0.91 | 1.00 |
| AA | rs11574110 |  | A | 0.02 | 0.02 | 963 | 1.07 | 0.55 | 2.09 | 0.84 | 1.00 |
| Caucasian | rs11574110 |  | A | 0.00 | 0.00 | 900 | NA | NA | NA | NA | 1.00 |
| AA | rs11574105 |  | A | 0.04 | 0.05 | 968 | 0.78 | 0.51 | 1.19 | 0.25 | 1.00 |
| Caucasian | rs11574105 |  | A | 0.01 | 0.00 | 921 | 1.54 | 0.44 | 5.39 | 0.50 | 1.00 |
| AA | rs12314197 |  | C | 0.19 | 0.23 | 970 | 0.85 | 0.68 | 1.06 | 0.14 | 0.99 |
| Caucasian | rs12314197 |  | C | 0.01 | 0.01 | 914 | 2.13 | 0.71 | 6.41 | 0.18 | 0.99 |
| AA | rs7962898 | *ApaI* | T | 0.39 | 0.34 | 967 | 1.20 | 0.99 | 1.45 | 0.07 | 0.91 |
| Caucasian | rs7962898 | *ApaI* | C | 0.48 | 0.48 | 904 | 1.04 | 0.87 | 1.25 | 0.69 | 1.00 |
| AA | rs7967152 |  | A | 0.38 | 0.39 | 967 | 0.96 | 0.80 | 1.16 | 0.66 | 1.00 |
| Caucasian | rs7967152 |  | A | 0.47 | 0.47 | 902 | 1.00 | 0.83 | 1.21 | 0.98 | 1.00 |
| AA | rs2239185 |  | C | 0.41 | 0.43 | 968 | 0.94 | 0.79 | 1.13 | 0.53 | 1.00 |
| Caucasian | rs2239185 |  | C | 0.47 | 0.47 | 907 | 1.04 | 0.86 | 1.25 | 0.71 | 1.00 |
| AA | rs7971418 |  | C | 0.43 | 0.45 | 970 | 0.94 | 0.78 | 1.13 | 0.48 | 1.00 |
| Caucasian | rs7971418 |  | C | 0.46 | 0.47 | 900 | 0.98 | 0.81 | 1.18 | 0.80 | 1.00 |
| AA | rs7975128 | *BsmI* | T | 0.30 | 0.27 | 970 | 1.14 | 0.93 | 1.39 | 0.22 | 1.00 |
| Caucasian | rs7975128 | *BsmI* | T | 0.38 | 0.39 | 923 | 0.90 | 0.74 | 1.09 | 0.28 | 1.00 |
| AA | rs11168264 |  | C | 0.20 | 0.25 | 962 | 0.80 | 0.64 | 1.00 | 0.05 | 0.87 |
| Caucasian | rs11168264 |  | C | 0.01 | 0.01 | 909 | 1.80 | 0.71 | 4.60 | 0.22 | 1.00 |
| AA | rs7966569 |  | C | 0.03 | 0.04 | 970 | 0.83 | 0.51 | 1.35 | 0.46 | 1.00 |
| Caucasian | rs7966569 |  | C | 0.00 | 0.00 | 899 | NA | NA | NA | NA | 1.00 |
| AA | rs7305032 |  | C | 0.29 | 0.29 | 969 | 1.00 | 0.83 | 1.22 | 0.97 | 1.00 |
| Caucasian | rs7305032 |  | C | 0.41 | 0.41 | 908 | 1.02 | 0.85 | 1.23 | 0.81 | 1.00 |
| AA | rs11574087 |  | T | 0.03 | 0.03 | 970 | 0.93 | 0.55 | 1.60 | 0.81 | 1.00 |
| Caucasian | rs11574087 |  | T | 0.00 | 0.00 | 912 | 0.00 | 0.00 | inf | 1.00 | 1.00 |
| Caucasian | rs11168266 |  | G | 0.44 | 0.43 | 900 | 1.05 | 0.87 | 1.26 | 0.63 | 1.00 |
| AA | rs11168267 |  | T | 0.08 | 0.07 | 971 | 1.23 | 0.87 | 1.75 | 0.24 | 1.00 |
| Caucasian | rs11168267 |  | T | 0.08 | 0.09 | 909 | 0.98 | 0.71 | 1.35 | 0.89 | 1.00 |
| AA | rs11168268 |  | C | 0.35 | 0.37 | 968 | 0.98 | 0.81 | 1.18 | 0.82 | 1.00 |
| Caucasian | rs11168268 |  | C | 0.43 | 0.42 | 876 | 1.05 | 0.87 | 1.26 | 0.61 | 1.00 |
| AA | rs12308082 |  | T | 0.07 | 0.09 | 970 | 0.78 | 0.56 | 1.08 | 0.14 | 0.99 |
| Caucasian | rs12308082 |  | T | 0.01 | 0.00 | 916 | 2.14 | 0.49 | 9.29 | 0.31 | 1.00 |
| AA | rs2853560 |  | T | 0.04 | 0.04 | 971 | 1.17 | 0.73 | 1.85 | 0.52 | 1.00 |
| Caucasian | rs2853560 |  | T | 0.00 | 0.00 | 928 | NA | NA | NA | NA | 1.00 |
| AA | rs2248098 |  | C | 0.52 | 0.48 | 964 | 1.15 | 0.96 | 1.38 | 0.14 | 0.99 |
| Caucasian | rs2248098 |  | C | 0.50 | 0.50 | 912 | 1.00 | 0.84 | 1.20 | 0.97 | 1.00 |
| AA | rs987849 |  | C | 0.25 | 0.25 | 964 | 0.99 | 0.81 | 1.22 | 0.93 | 1.00 |
| Caucasian | rs987849 |  | C | 0.44 | 0.45 | 867 | 0.98 | 0.82 | 1.17 | 0.80 | 1.00 |
| AA | rs2239182 |  | A | 0.42 | 0.41 | 971 | 1.08 | 0.90 | 1.31 | 0.41 | 1.00 |
| Caucasian | rs2239182 |  | G | 0.50 | 0.50 | 893 | 0.98 | 0.81 | 1.17 | 0.80 | 1.00 |
| AA | rs2107301 |  | T | 0.16 | 0.17 | 971 | 0.97 | 0.75 | 1.24 | 0.79 | 1.00 |
| Caucasian | rs2107301 |  | T | 0.28 | 0.28 | 887 | 1.00 | 0.81 | 1.23 | 1.00 | 1.00 |
| AA | rs1540339 |  | A | 0.21 | 0.22 | 965 | 0.93 | 0.74 | 1.17 | 0.54 | 1.00 |
| Caucasian | rs1540339 |  | A | 0.38 | 0.38 | 924 | 0.97 | 0.80 | 1.17 | 0.73 | 1.00 |
| AA | rs2239179 |  | G | 0.36 | 0.33 | 963 | 1.08 | 0.89 | 1.32 | 0.42 | 1.00 |
| Caucasian | rs2239179 |  | G | 0.42 | 0.44 | 864 | 0.91 | 0.75 | 1.10 | 0.31 | 1.00 |
| AA | rs11574070 |  | T | 0.07 | 0.07 | 963 | 1.20 | 0.83 | 1.72 | 0.34 | 1.00 |
| Caucasian | rs11574070 |  | T | 0.00 | 0.00 | 910 | 0.27 | 0.03 | 2.60 | 0.25 | 1.00 |
| AA | rs11574065 |  | T | 0.03 | 0.02 | 971 | 1.21 | 0.67 | 2.19 | 0.52 | 1.00 |
| Caucasian | rs11574065 |  | T | 0.00 | 0.00 | 916 | NA | NA | NA | NA | 1.00 |
| AA | rs12717991 |  | A | 0.30 | 0.30 | 971 | 1.02 | 0.84 | 1.24 | 0.85 | 1.00 |
| Caucasian | rs12717991 |  | A | 0.38 | 0.38 | 910 | 0.98 | 0.81 | 1.19 | 0.85 | 1.00 |
| AA | rs2189480 |  | A | 0.36 | 0.37 | 956 | 0.98 | 0.81 | 1.19 | 0.84 | 1.00 |
| Caucasian | rs2189480 |  | A | 0.37 | 0.34 | 914 | 1.04 | 0.85 | 1.26 | 0.72 | 1.00 |
| AA | rs3819545 |  | C | 0.24 | 0.26 | 971 | 0.91 | 0.73 | 1.12 | 0.36 | 1.00 |
| Caucasian | rs3819545 |  | C | 0.41 | 0.39 | 900 | 1.02 | 0.85 | 1.24 | 0.82 | 1.00 |
| AA | rs3782905 |  | G | 0.24 | 0.23 | 968 | 1.01 | 0.82 | 1.26 | 0.90 | 1.00 |
| Caucasian | rs3782905 |  | G | 0.31 | 0.34 | 925 | 0.87 | 0.71 | 1.06 | 0.15 | 0.96 |
| AA | rs11574050 |  | T | 0.08 | 0.09 | 971 | 0.87 | 0.63 | 1.21 | 0.41 | 1.00 |
| Caucasian | rs11574050 |  | T | 0.05 | 0.05 | 911 | 0.94 | 0.62 | 1.43 | 0.79 | 1.00 |
| AA | rs10783218 |  | T | 0.16 | 0.19 | 968 | 0.82 | 0.65 | 1.05 | 0.11 | 0.98 |
| Caucasian | rs10783218 |  | T | 0.04 | 0.03 | 908 | 1.62 | 0.95 | 2.78 | 0.08 | 0.97 |
| AA | rs10735810 | *FokI* | T | 0.22 | 0.22 | 969 | 0.92 | 0.74 | 1.15 | 0.47 | 1.00 |
| Caucasian | rs10735810 | *FokI* | T | 0.38 | 0.36 | 897 | 1.12 | 0.92 | 1.36 | 0.27 | 1.00 |
| AA | rs2408876 |  | G | 0.46 | 0.47 | 967 | 0.94 | 0.79 | 1.13 | 0.52 | 1.00 |
| Caucasian | rs2408876 |  | G | 0.40 | 0.41 | 875 | 0.93 | 0.76 | 1.13 | 0.47 | 1.00 |
| AA | rs2254210 |  | T | 0.35 | 0.34 | 971 | 1.04 | 0.86 | 1.26 | 0.68 | 1.00 |
| Caucasian | rs2254210 |  | T | 0.34 | 0.36 | 905 | 0.96 | 0.79 | 1.16 | 0.67 | 1.00 |
| AA | rs11574044 |  | G | 0.27 | 0.25 | 966 | 1.10 | 0.90 | 1.36 | 0.35 | 1.00 |
| Caucasian | rs11574044 |  | G | 0.14 | 0.17 | 900 | 0.85 | 0.65 | 1.10 | 0.21 | 0.98 |
| AA | rs11574041 |  | A | 0.09 | 0.09 | 971 | 1.00 | 0.73 | 1.37 | 0.99 | 1.00 |
| Caucasian | rs11574041 |  | A | 0.00 | 0.00 | 897 | NA | NA | NA | NA | 1.00 |
| AA | rs2238136 |  | A | 0.09 | 0.09 | 971 | 0.87 | 0.62 | 1.20 | 0.39 | 1.00 |
| Caucasian | rs2238136 |  | A | 0.26 | 0.27 | 900 | 0.94 | 0.76 | 1.16 | 0.56 | 1.00 |
| AA | rs2238135 |  | C | 0.30 | 0.31 | 970 | 0.95 | 0.78 | 1.15 | 0.59 | 1.00 |
| Caucasian | rs2238135 |  | C | 0.25 | 0.27 | 922 | 0.93 | 0.75 | 1.15 | 0.51 | 1.00 |
| AA | rs2853564 |  | C | 0.13 | 0.12 | 971 | 0.97 | 0.74 | 1.27 | 0.82 | 1.00 |
| Caucasian | rs2853564 |  | C | 0.39 | 0.38 | 911 | 1.03 | 0.85 | 1.26 | 0.73 | 1.00 |
| AA | rs2853559 |  | T | 0.18 | 0.18 | 965 | 1.00 | 0.79 | 1.27 | 0.99 | 1.00 |
| Caucasian | rs2853559 |  | T | 0.38 | 0.38 | 836 | 0.96 | 0.78 | 1.16 | 0.65 | 1.00 |
| AA | rs11168287 |  | C | 0.29 | 0.30 | 970 | 0.99 | 0.81 | 1.21 | 0.93 | 1.00 |
| Caucasian | rs11168287 |  | T | 0.49 | 0.49 | 891 | 0.98 | 0.81 | 1.19 | 0.86 | 1.00 |
| AA | rs4328262 |  | C | 0.32 | 0.31 | 963 | 1.03 | 0.85 | 1.25 | 0.78 | 1.00 |
| Caucasian | rs4328262 |  | C | 0.43 | 0.44 | 892 | 0.97 | 0.80 | 1.18 | 0.75 | 1.00 |
| AA | rs4334089 |  | C | 0.40 | 0.38 | 970 | 1.07 | 0.89 | 1.28 | 0.48 | 1.00 |
| Caucasian | rs4334089 |  | T | 0.28 | 0.26 | 903 | 1.05 | 0.85 | 1.30 | 0.66 | 1.00 |
| AA | rs3890733 |  | A | 0.15 | 0.15 | 950 | 0.97 | 0.75 | 1.26 | 0.82 | 1.00 |
| Caucasian | rs3890733 |  | A | 0.31 | 0.31 | 849 | 1.01 | 0.82 | 1.24 | 0.96 | 1.00 |
| AA | rs7302235 |  | G | 0.48 | 0.50 | 957 | 0.94 | 0.78 | 1.12 | 0.47 | 1.00 |
| Caucasian | rs7302235 |  | G | 0.27 | 0.27 | 844 | 1.00 | 0.81 | 1.24 | 0.99 | 1.00 |
| AA | rs7136534 |  | A | 0.10 | 0.11 | 967 | 0.94 | 0.70 | 1.26 | 0.68 | 1.00 |
| Caucasian | rs7136534 |  | A | 0.27 | 0.24 | 898 | 1.11 | 0.89 | 1.38 | 0.35 | 0.99 |
| AA | rs11574002 |  | G | 0.03 | 0.03 | 969 | 1.07 | 0.65 | 1.78 | 0.78 | 1.00 |
| Caucasian | rs11574002 |  | G | 0.00 | 0.00 | 912 | NA | NA | NA | NA | 1.00 |
| **(B) Males** |  |  |  |  |  |  |  |  |  |  |  |
| AA | rs11574141 |  | C | 0.06 | 0.07 | 647 | 0.87 | 0.56 | 1.35 | 0.53 | 1.00 |
| Caucasians | rs11574141 |  | C | 0.00 | 0.00 | 1213 | 1.92 | 0.19 | 18.93 | 0.58 | 1.00 |
| AA | rs2853563 |  | A | 0.15 | 0.17 | 646 | 0.87 | 0.64 | 1.18 | 0.36 | 1.00 |
| Caucasians | rs2853563 |  | A | 0.07 | 0.05 | 1190 | 1.33 | 0.92 | 1.91 | 0.13 | 0.95 |
| AA | rs7954412 |  | G | 0.05 | 0.08 | 647 | 0.67 | 0.43 | 1.03 | 0.07 | 0.91 |
| Caucasians | rs7954412 |  | G | 0.00 | 0.00 | 1213 | 4.10 | 0.48 | 34.77 | 0.20 | 0.99 |
| AA | rs3858733 |  | C | 0.01 | 0.00 | 647 | 3.94 | 0.84 | 18.46 | 0.08 | 0.95 |
| Caucasians | rs3858733 |  | C | 0.06 | 0.05 | 1211 | 1.18 | 0.81 | 1.71 | 0.39 | 1.00 |
| AA | rs739837 |  | C | 0.41 | 0.41 | 646 | 1.01 | 0.81 | 1.26 | 0.96 | 1.00 |
| Caucasians | rs739837 |  | C | 0.46 | 0.48 | 1207 | 0.91 | 0.77 | 1.07 | 0.27 | 1.00 |
| AA | rs731236 | *TaqI* | C | 0.30 | 0.31 | 644 | 0.92 | 0.72 | 1.17 | 0.49 | 1.00 |
| Caucasians | rs731236 | *TaqI* | C | 0.39 | 0.37 | 1199 | 1.08 | 0.91 | 1.28 | 0.37 | 1.00 |
| AA | rs11574114 |  | A | 0.13 | 0.13 | 647 | 1.09 | 0.78 | 1.51 | 0.62 | 1.00 |
| Caucasians | rs11574114 |  | A | 0.07 | 0.05 | 1206 | 1.27 | 0.87 | 1.83 | 0.21 | 0.99 |
| AA | rs11574110 |  | A | 0.02 | 0.01 | 632 | 1.79 | 0.66 | 4.85 | 0.25 | 1.00 |
| Caucasians | rs11574110 |  | A | 0.00 | 0.00 | 1204 | NA | NA | NA | NA | 1.00 |
| AA | rs11574105 |  | A | 0.04 | 0.04 | 644 | 0.96 | 0.54 | 1.72 | 0.90 | 1.00 |
| Caucasians | rs11574105 |  | A | 0.00 | 0.00 | 1204 | 1.44 | 0.26 | 7.99 | 0.68 | 1.00 |
| AA | rs12314197 |  | C | 0.20 | 0.22 | 647 | 0.90 | 0.69 | 1.18 | 0.44 | 1.00 |
| Caucasians | rs12314197 |  | C | 0.00 | 0.00 | 1212 | 2.19 | 0.44 | 10.81 | 0.34 | 1.00 |
| AA | rs7962898 | *ApaI* | T | 0.39 | 0.36 | 640 | 1.11 | 0.88 | 1.39 | 0.37 | 1.00 |
| Caucasians | rs7962898 | *ApaI* | C | 0.46 | 0.49 | 1182 | 0.91 | 0.77 | 1.08 | 0.28 | 1.00 |
| AA | rs7967152 |  | A | 0.38 | 0.38 | 642 | 1.00 | 0.80 | 1.26 | 0.98 | 1.00 |
| Caucasians | rs7967152 |  | A | 0.45 | 0.48 | 1205 | 0.90 | 0.76 | 1.06 | 0.20 | 0.99 |
| AA | rs2239185 |  | C | 0.41 | 0.43 | 647 | 0.90 | 0.73 | 1.12 | 0.36 | 1.00 |
| Caucasians | rs2239185 |  | C | 0.46 | 0.48 | 1196 | 0.91 | 0.77 | 1.08 | 0.27 | 1.00 |
| AA | rs7971418 |  | C | 0.42 | 0.44 | 647 | 0.89 | 0.71 | 1.11 | 0.30 | 1.00 |
| Caucasians | rs7971418 |  | C | 0.44 | 0.48 | 1182 | 0.87 | 0.73 | 1.03 | 0.10 | 0.90 |
| AA | rs7975128 | *BsmI* | T | 0.31 | 0.29 | 646 | 1.10 | 0.87 | 1.39 | 0.44 | 1.00 |
| Caucasians | rs7975128 | *BsmI* | T | 0.40 | 0.38 | 1211 | 1.10 | 0.93 | 1.30 | 0.28 | 1.00 |
| AA | rs11168264 |  | C | 0.22 | 0.23 | 638 | 0.96 | 0.74 | 1.26 | 0.79 | 1.00 |
| Caucasians | rs11168264 |  | C | 0.00 | 0.00 | 1201 | 1.29 | 0.33 | 5.09 | 0.71 | 1.00 |
| AA | rs7966569 |  | C | 0.04 | 0.03 | 647 | 1.45 | 0.78 | 2.68 | 0.24 | 1.00 |
| Caucasians | rs7966569 |  | C | 0.00 | 0.00 | 1205 | NA | NA | NA | NA | 1.00 |
| AA | rs7305032 |  | C | 0.29 | 0.31 | 646 | 0.93 | 0.73 | 1.18 | 0.55 | 1.00 |
| Caucasians | rs7305032 |  | C | 0.40 | 0.44 | 1208 | 0.87 | 0.73 | 1.03 | 0.11 | 0.92 |
| AA | rs11574087 |  | T | 0.02 | 0.03 | 645 | 0.68 | 0.32 | 1.43 | 0.31 | 1.00 |
| Caucasians | rs11574087 |  | T | 0.00 | 0.00 | 1213 | NA | NA | NA | NA | 1.00 |
| Caucasians | rs11168266 |  | G | 0.41 | 0.44 | 1199 | 0.88 | 0.74 | 1.04 | 0.13 | 0.95 |
| AA | rs11168267 |  | T | 0.07 | 0.06 | 646 | 1.16 | 0.75 | 1.80 | 0.51 | 1.00 |
| Caucasians | rs11168267 |  | T | 0.10 | 0.10 | 1211 | 1.02 | 0.76 | 1.35 | 0.91 | 1.00 |
| AA | rs11168268 |  | C | 0.38 | 0.35 | 646 | 1.09 | 0.88 | 1.36 | 0.44 | 1.00 |
| Caucasians | rs11168268 |  | C | 0.41 | 0.44 | 1185 | 0.88 | 0.74 | 1.04 | 0.14 | 0.95 |
| AA | rs12308082 |  | T | 0.07 | 0.10 | 647 | 0.71 | 0.47 | 1.06 | 0.09 | 0.97 |
| Caucasians | rs12308082 |  | T | 0.00 | 0.00 | 1212 | 1.45 | 0.13 | 16.29 | 0.77 | 1.00 |
| AA | rs2853560 |  | T | 0.04 | 0.04 | 646 | 1.00 | 0.56 | 1.79 | 0.99 | 1.00 |
| Caucasians | rs2853560 |  | T | 0.00 | 0.00 | 1212 | NA | NA | NA | 1.00 | 1.00 |
| AA | rs2248098 |  | T | 0.49 | 0.50 | 644 | 0.96 | 0.77 | 1.19 | 0.68 | 1.00 |
| Caucasians | rs2248098 |  | T | 0.48 | 0.51 | 1209 | 0.89 | 0.75 | 1.05 | 0.15 | 0.98 |
| AA | rs987849 |  | C | 0.26 | 0.26 | 639 | 0.95 | 0.74 | 1.23 | 0.71 | 1.00 |
| Caucasians | rs987849 |  | C | 0.43 | 0.48 | 1171 | 0.86 | 0.73 | 1.01 | 0.07 | 0.75 |
| AA | rs2239182 |  | A | 0.39 | 0.39 | 644 | 1.02 | 0.82 | 1.27 | 0.89 | 1.00 |
| Caucasians | rs2239182 |  | G | 0.50 | 0.46 | 1199 | 1.15 | 0.97 | 1.35 | 0.11 | 0.92 |
| AA | rs2107301 |  | T | 0.17 | 0.15 | 647 | 1.18 | 0.88 | 1.59 | 0.27 | 1.00 |
| Caucasians | rs2107301 |  | T | 0.30 | 0.29 | 1193 | 0.99 | 0.83 | 1.19 | 0.93 | 1.00 |
| AA | rs1540339 |  | A | 0.21 | 0.19 | 646 | 1.11 | 0.85 | 1.46 | 0.45 | 1.00 |
| Caucasians | rs1540339 |  | A | 0.37 | 0.38 | 1204 | 0.94 | 0.79 | 1.12 | 0.49 | 1.00 |
| AA | rs2239179 |  | G | 0.39 | 0.36 | 638 | 1.11 | 0.89 | 1.40 | 0.36 | 1.00 |
| Caucasians | rs2239179 |  | G | 0.43 | 0.40 | 1160 | 1.13 | 0.95 | 1.34 | 0.18 | 0.99 |
| AA | rs11574070 |  | T | 0.08 | 0.09 | 639 | 1.00 | 0.67 | 1.49 | 0.99 | 1.00 |
| Caucasians | rs11574070 |  | T | 0.00 | 0.00 | 1210 | NA | NA | NA | NA | 1.00 |
| AA | rs11574065 |  | T | 0.04 | 0.02 | 645 | 2.32 | 1.13 | 4.78 | 0.02 | 0.52 |
| Caucasians | rs11574065 |  | T | 0.00 | 0.00 | 1213 | NA | NA | NA | NA | 1.00 |
| AA | rs12717991 |  | A | 0.30 | 0.29 | 647 | 1.02 | 0.80 | 1.30 | 0.87 | 1.00 |
| Caucasians | rs12717991 |  | A | 0.38 | 0.38 | 1206 | 0.98 | 0.83 | 1.16 | 0.83 | 1.00 |
| AA | rs2189480 |  | A | 0.34 | 0.35 | 640 | 0.94 | 0.75 | 1.18 | 0.60 | 1.00 |
| Caucasians | rs2189480 |  | A | 0.36 | 0.36 | 1195 | 0.94 | 0.79 | 1.12 | 0.46 | 1.00 |
| AA | rs3819545 |  | C | 0.25 | 0.24 | 642 | 1.04 | 0.80 | 1.35 | 0.78 | 1.00 |
| Caucasians | rs3819545 |  | C | 0.40 | 0.40 | 1200 | 0.93 | 0.78 | 1.11 | 0.41 | 1.00 |
| AA | rs3782905 |  | G | 0.25 | 0.23 | 644 | 1.11 | 0.86 | 1.44 | 0.42 | 1.00 |
| Caucasians | rs3782905 |  | G | 0.32 | 0.31 | 1207 | 1.11 | 0.93 | 1.32 | 0.25 | 1.00 |
| AA | rs11574050 |  | T | 0.11 | 0.07 | 646 | 1.67 | 1.13 | 2.47 | 0.01 | 0.29 |
| Caucasians | rs11574050 |  | T | 0.06 | 0.05 | 1213 | 1.27 | 0.88 | 1.85 | 0.21 | 0.99 |
| AA | rs10783218 |  | T | 0.17 | 0.19 | 641 | 0.90 | 0.68 | 1.20 | 0.46 | 1.00 |
| Caucasians | rs10783218 |  | T | 0.03 | 0.02 | 1204 | 1.33 | 0.79 | 2.24 | 0.28 | 1.00 |
| AA | rs10735810 | *FokI* | T | 0.22 | 0.22 | 646 | 1.06 | 0.81 | 1.39 | 0.69 | 1.00 |
| Caucasians | rs10735810 | *FokI* | T | 0.36 | 0.39 | 1209 | 0.91 | 0.77 | 1.08 | 0.27 | 1.00 |
| AA | rs2408876 |  | G | 0.48 | 0.49 | 638 | 0.95 | 0.75 | 1.19 | 0.63 | 1.00 |
| Caucasians | rs2408876 |  | G | 0.43 | 0.45 | 1151 | 0.95 | 0.79 | 1.13 | 0.53 | 1.00 |
| AA | rs2254210 |  | T | 0.30 | 0.30 | 646 | 0.97 | 0.76 | 1.23 | 0.80 | 1.00 |
| Caucasians | rs2254210 |  | T | 0.35 | 0.30 | 1209 | 1.30 | 1.08 | 1.57 | 0.01 | 0.11 |
| AA | rs11574044 |  | G | 0.27 | 0.24 | 635 | 1.18 | 0.91 | 1.53 | 0.20 | 1.00 |
| Caucasians | rs11574044 |  | G | 0.17 | 0.17 | 1202 | 0.95 | 0.76 | 1.19 | 0.66 | 1.00 |
| AA | rs11574041 |  | A | 0.07 | 0.10 | 646 | 0.73 | 0.49 | 1.08 | 0.12 | 0.99 |
| Caucasians | rs11574041 |  | A | 0.00 | 0.00 | 1200 | NA | NA | NA | NA | 1.00 |
| AA | rs2238136 |  | A | 0.11 | 0.10 | 646 | 1.04 | 0.73 | 1.49 | 0.82 | 1.00 |
| Caucasians | rs2238136 |  | A | 0.27 | 0.28 | 1207 | 0.97 | 0.80 | 1.16 | 0.71 | 1.00 |
| AA | rs2238135 |  | C | 0.32 | 0.36 | 646 | 0.85 | 0.67 | 1.07 | 0.17 | 1.00 |
| Caucasians | rs2238135 |  | C | 0.26 | 0.27 | 1204 | 0.97 | 0.80 | 1.17 | 0.74 | 1.00 |
| AA | rs2853564 |  | C | 0.13 | 0.11 | 647 | 1.19 | 0.84 | 1.67 | 0.33 | 1.00 |
| Caucasians | rs2853564 |  | C | 0.37 | 0.33 | 1210 | 1.22 | 1.02 | 1.46 | 0.03 | 0.43 |
| AA | rs2853559 |  | T | 0.18 | 0.15 | 640 | 1.27 | 0.94 | 1.71 | 0.12 | 0.99 |
| Caucasians | rs2853559 |  | T | 0.37 | 0.33 | 1123 | 1.18 | 0.99 | 1.42 | 0.06 | 0.74 |
| AA | rs11168287 |  | C | 0.26 | 0.29 | 646 | 0.83 | 0.65 | 1.06 | 0.14 | 1.00 |
| Caucasians | rs11168287 |  | T | 0.48 | 0.46 | 1205 | 1.13 | 0.96 | 1.34 | 0.15 | 0.98 |
| AA | rs4328262 |  | C | 0.30 | 0.33 | 644 | 0.91 | 0.72 | 1.15 | 0.43 | 1.00 |
| Caucasians | rs4328262 |  | C | 0.44 | 0.45 | 1202 | 0.94 | 0.79 | 1.11 | 0.47 | 1.00 |
| AA | rs4334089 |  | C | 0.39 | 0.39 | 641 | 0.99 | 0.79 | 1.24 | 0.95 | 1.00 |
| Caucasians | rs4334089 |  | T | 0.27 | 0.28 | 1206 | 0.93 | 0.77 | 1.12 | 0.44 | 1.00 |
| AA | rs3890733 |  | A | 0.16 | 0.13 | 629 | 1.27 | 0.93 | 1.75 | 0.13 | 0.99 |
| Caucasians | rs3890733 |  | A | 0.30 | 0.28 | 1162 | 1.13 | 0.93 | 1.37 | 0.21 | 0.99 |
| AA | rs7302235 |  | G | 0.49 | 0.49 | 632 | 1.03 | 0.83 | 1.28 | 0.78 | 1.00 |
| Caucasians | rs7302235 |  | G | 0.27 | 0.29 | 1158 | 0.92 | 0.77 | 1.10 | 0.37 | 1.00 |
| AA | rs7136534 |  | A | 0.11 | 0.11 | 643 | 0.89 | 0.63 | 1.27 | 0.52 | 1.00 |
| Caucasians | rs7136534 |  | A | 0.25 | 0.28 | 1207 | 0.88 | 0.74 | 1.06 | 0.19 | 0.99 |
| AA | rs11574002 |  | G | 0.04 | 0.04 | 644 | 1.09 | 0.60 | 1.97 | 0.78 | 1.00 |
| Caucasians | rs11574002 |  | G | 0.00 | 0.00 | 1213 | 1.60 | 0.14 | 18.60 | 0.71 | 1.00 |
| AA, African American; SNP, single nucleotide polymorphism; RFLP, restriction fragment length polymorphism; Freq., allele frequency; Inds, number of individuals included for each analysis; L95, lower boundary 95% confidence interval; U95, upper boundary 95% confidence interval; NA, not applicable due to monomorphic allele; inf, infinite.  *OR and p-value adjusted for age, gender and West African ancestry (ancestry only adjusted for in African American group). | | | | | | | | | | | |
