# Supplementary material for: Genetic Associations in the Vitamin D Receptor and Colorectal Cancer in African Americans and Caucasians
Source: PLoS One. 2011 Oct 27;6(10):e26123. doi: 10.1371/journal.pone.0026123 (PMC3203108; doi:10.1371/journal.pone.0026123)
Supplement: Table S5 — VDR associations by anatomic site: (A) colon cancer (B) rectal cancer. (DOCX) [file pone.0026123.s007.docx]

| **Supplementary Table S5: *VDR* associations by anatomic site: (A) colon cancer (B) rectal cancer.** | | | | | | | | | | | |
| --- | --- | --- | --- | --- | --- | --- | --- | --- | --- | --- | --- |
| Ancestry | SNP | RFLP | Allele | Freq. cases | Freq. controls | Inds. | OR* | L95 | U95 | P-value* | Permuted p-value |
| **(A) Colon** |  |  |  |  |  |  |  |  |  |  |  |
| AA | rs11574141 |  | C | 0.07 | 0.08 | 1430 | 0.84 | 0.63 | 1.13 | 0.25 | 1.00 |
| Caucasian | rs11574141 |  | C | 0.00 | 0.00 | 1573 | 2.33 | 0.64 | 8.51 | 0.20 | 0.99 |
| AA | rs2853563 |  | A | 0.15 | 0.16 | 1422 | 0.95 | 0.77 | 1.17 | 0.64 | 1.00 |
| Caucasian | rs2853563 |  | A | 0.07 | 0.06 | 1548 | 1.19 | 0.89 | 1.60 | 0.24 | 1.00 |
| AA | rs7954412 |  | G | 0.06 | 0.07 | 1429 | 0.89 | 0.65 | 1.21 | 0.45 | 1.00 |
| Caucasian | rs7954412 |  | G | 0.00 | 0.00 | 1571 | 5.25 | 0.57 | 48.80 | 0.14 | 0.96 |
| AA | rs3858733 |  | C | 0.01 | 0.01 | 1430 | 1.00 | 0.48 | 2.10 | 1.00 | 1.00 |
| Caucasian | rs3858733 |  | C | 0.07 | 0.06 | 1569 | 1.10 | 0.82 | 1.47 | 0.54 | 1.00 |
| AA | rs739837 |  | C | 0.41 | 0.42 | 1427 | 0.97 | 0.84 | 1.13 | 0.73 | 1.00 |
| Caucasian | rs739837 |  | C | 0.47 | 0.47 | 1566 | 0.99 | 0.86 | 1.14 | 0.93 | 1.00 |
| AA | rs731236 | *TaqI* | C | 0.30 | 0.30 | 1423 | 0.97 | 0.82 | 1.15 | 0.71 | 1.00 |
| Caucasian | rs731236 | *TaqI* | C | 0.38 | 0.38 | 1549 | 1.02 | 0.88 | 1.18 | 0.78 | 1.00 |
| AA | rs11574114 |  | A | 0.15 | 0.15 | 1429 | 1.02 | 0.82 | 1.26 | 0.89 | 1.00 |
| Caucasian | rs11574114 |  | A | 0.07 | 0.06 | 1564 | 1.07 | 0.80 | 1.44 | 0.63 | 1.00 |
| AA | rs11574110 |  | A | 0.02 | 0.02 | 1408 | 1.38 | 0.78 | 2.43 | 0.27 | 1.00 |
| Caucasian | rs11574110 |  | A | 0.00 | 0.00 | 1557 | NA | NA | NA | NA | 1.00 |
| AA | rs11574105 |  | A | 0.04 | 0.05 | 1425 | 0.91 | 0.64 | 1.31 | 0.63 | 1.00 |
| Caucasian | rs11574105 |  | A | 0.00 | 0.00 | 1560 | 1.51 | 0.47 | 4.90 | 0.49 | 1.00 |
| AA | rs12314197 |  | C | 0.21 | 0.23 | 1429 | 0.93 | 0.78 | 1.12 | 0.46 | 1.00 |
| Caucasian | rs12314197 |  | C | 0.01 | 0.00 | 1571 | 1.65 | 0.62 | 4.40 | 0.31 | 1.00 |
| AA | rs7962898 | *ApaI* | T | 0.38 | 0.35 | 1420 | 1.12 | 0.96 | 1.31 | 0.16 | 1.00 |
| Caucasian | rs7962898 | *ApaI* | C | 0.48 | 0.48 | 1530 | 1.00 | 0.87 | 1.15 | 0.99 | 1.00 |
| AA | rs7967152 |  | A | 0.37 | 0.38 | 1423 | 0.96 | 0.82 | 1.12 | 0.60 | 1.00 |
| Caucasian | rs7967152 |  | A | 0.47 | 0.47 | 1564 | 0.99 | 0.86 | 1.14 | 0.85 | 1.00 |
| AA | rs2239185 |  | C | 0.40 | 0.43 | 1427 | 0.90 | 0.77 | 1.05 | 0.18 | 1.00 |
| Caucasian | rs2239185 |  | C | 0.47 | 0.47 | 1544 | 0.99 | 0.86 | 1.14 | 0.92 | 1.00 |
| AA | rs7971418 |  | C | 0.42 | 0.45 | 1429 | 0.90 | 0.77 | 1.05 | 0.17 | 1.00 |
| Caucasian | rs7971418 |  | C | 0.46 | 0.47 | 1536 | 0.94 | 0.82 | 1.09 | 0.43 | 1.00 |
| AA | rs7975128 | *BsmI* | T | 0.30 | 0.27 | 1428 | 1.10 | 0.93 | 1.30 | 0.26 | 1.00 |
| Caucasian | rs7975128 | *BsmI* | T | 0.39 | 0.39 | 1572 | 1.01 | 0.88 | 1.16 | 0.90 | 1.00 |
| AA | rs11168264 |  | C | 0.23 | 0.24 | 1411 | 0.93 | 0.78 | 1.12 | 0.45 | 1.00 |
| Caucasian | rs11168264 |  | C | 0.01 | 0.01 | 1557 | 1.47 | 0.64 | 3.38 | 0.37 | 1.00 |
| AA | rs7966569 |  | C | 0.03 | 0.04 | 1429 | 0.86 | 0.56 | 1.31 | 0.47 | 1.00 |
| Caucasian | rs7966569 |  | C | 0.00 | 0.00 | 1562 | NA | NA | NA | NA | 1.00 |
| AA | rs7305032 |  | C | 0.28 | 0.30 | 1427 | 0.95 | 0.80 | 1.12 | 0.51 | 1.00 |
| Caucasian | rs7305032 |  | C | 0.41 | 0.42 | 1566 | 0.98 | 0.85 | 1.13 | 0.74 | 1.00 |
| AA | rs11574087 |  | T | 0.02 | 0.03 | 1427 | 0.85 | 0.53 | 1.37 | 0.51 | 1.00 |
| Caucasian | rs11574087 |  | T | 0.00 | 0.00 | 1573 | NA | NA | NA | NA | 1.00 |
| Caucasian | rs11168266 |  | G | 0.43 | 0.43 | 1556 | 0.99 | 0.86 | 1.14 | 0.92 | 1.00 |
| AA | rs11168267 |  | T | 0.08 | 0.07 | 1429 | 1.16 | 0.86 | 1.56 | 0.33 | 1.00 |
| Caucasian | rs11168267 |  | T | 0.09 | 0.09 | 1571 | 0.92 | 0.72 | 1.18 | 0.53 | 1.00 |
| AA | rs11168268 |  | C | 0.36 | 0.36 | 1426 | 1.02 | 0.87 | 1.19 | 0.85 | 1.00 |
| Caucasian | rs11168268 |  | C | 0.43 | 0.43 | 1525 | 1.00 | 0.87 | 1.15 | 0.99 | 1.00 |
| AA | rs12308082 |  | T | 0.08 | 0.09 | 1429 | 0.80 | 0.61 | 1.05 | 0.10 | 0.98 |
| Caucasian | rs12308082 |  | T | 0.00 | 0.00 | 1572 | 1.89 | 0.52 | 6.85 | 0.34 | 1.00 |
| AA | rs2853560 |  | T | 0.04 | 0.04 | 1429 | 1.19 | 0.81 | 1.74 | 0.38 | 1.00 |
| Caucasian | rs2853560 |  | T | 0.00 | 0.00 | 1573 | NA | NA | NA | NA | 1.00 |
| AA | rs2248098 |  | C | 0.51 | 0.49 | 1421 | 1.09 | 0.94 | 1.27 | 0.27 | 1.00 |
| Caucasian | rs2248098 |  | T | 0.50 | 0.50 | 1571 | 0.98 | 0.86 | 1.13 | 0.81 | 1.00 |
| AA | rs987849 |  | C | 0.24 | 0.25 | 1415 | 0.93 | 0.78 | 1.11 | 0.40 | 1.00 |
| Caucasian | rs987849 |  | C | 0.45 | 0.46 | 1516 | 0.94 | 0.82 | 1.08 | 0.41 | 1.00 |
| AA | rs2239182 |  | A | 0.40 | 0.40 | 1427 | 0.98 | 0.84 | 1.15 | 0.83 | 1.00 |
| Caucasian | rs2239182 |  | G | 0.49 | 0.48 | 1549 | 1.04 | 0.91 | 1.20 | 0.57 | 1.00 |
| AA | rs2107301 |  | T | 0.17 | 0.16 | 1430 | 1.08 | 0.88 | 1.32 | 0.48 | 1.00 |
| Caucasian | rs2107301 |  | T | 0.30 | 0.29 | 1545 | 1.01 | 0.87 | 1.18 | 0.90 | 1.00 |
| AA | rs1540339 |  | A | 0.22 | 0.21 | 1424 | 1.01 | 0.84 | 1.22 | 0.89 | 1.00 |
| Caucasian | rs1540339 |  | A | 0.37 | 0.38 | 1566 | 0.94 | 0.81 | 1.09 | 0.43 | 1.00 |
| AA | rs2239179 |  | G | 0.36 | 0.34 | 1415 | 1.10 | 0.93 | 1.29 | 0.26 | 1.00 |
| Caucasian | rs2239179 |  | G | 0.43 | 0.42 | 1489 | 1.04 | 0.90 | 1.20 | 0.61 | 1.00 |
| AA | rs11574070 |  | T | 0.08 | 0.07 | 1415 | 1.20 | 0.90 | 1.60 | 0.21 | 1.00 |
| Caucasian | rs11574070 |  | T | 0.00 | 0.00 | 1565 | 0.59 | 0.05 | 6.86 | 0.67 | 1.00 |
| AA | rs11574065 |  | T | 0.03 | 0.02 | 1429 | 1.47 | 0.91 | 2.38 | 0.12 | 0.99 |
| Caucasian | rs11574065 |  | T | 0.00 | 0.00 | 1573 | NA | NA | NA | NA | 1.00 |
| AA | rs12717991 |  | A | 0.31 | 0.30 | 1430 | 1.08 | 0.91 | 1.27 | 0.38 | 1.00 |
| Caucasian | rs12717991 |  | A | 0.38 | 0.38 | 1564 | 0.95 | 0.82 | 1.10 | 0.46 | 1.00 |
| AA | rs2189480 |  | A | 0.36 | 0.36 | 1410 | 1.00 | 0.86 | 1.18 | 0.96 | 1.00 |
| Caucasian | rs2189480 |  | A | 0.36 | 0.35 | 1551 | 0.99 | 0.85 | 1.14 | 0.86 | 1.00 |
| AA | rs3819545 |  | C | 0.24 | 0.25 | 1425 | 0.93 | 0.78 | 1.11 | 0.42 | 1.00 |
| Caucasian | rs3819545 |  | C | 0.39 | 0.40 | 1555 | 0.92 | 0.79 | 1.06 | 0.25 | 1.00 |
| AA | rs3782905 |  | G | 0.24 | 0.23 | 1425 | 1.05 | 0.88 | 1.26 | 0.59 | 1.00 |
| Caucasian | rs3782905 |  | G | 0.31 | 0.32 | 1567 | 0.98 | 0.84 | 1.14 | 0.77 | 1.00 |
| AA | rs11574050 |  | T | 0.09 | 0.08 | 1429 | 1.13 | 0.86 | 1.47 | 0.37 | 1.00 |
| Caucasian | rs11574050 |  | T | 0.06 | 0.05 | 1572 | 1.13 | 0.83 | 1.54 | 0.44 | 1.00 |
| AA | rs10783218 |  | T | 0.17 | 0.19 | 1421 | 0.86 | 0.71 | 1.05 | 0.14 | 1.00 |
| Caucasian | rs10783218 |  | T | 0.03 | 0.02 | 1563 | 1.43 | 0.92 | 2.21 | 0.11 | 0.91 |
| AA | rs10735810 | *FokI* | T | 0.22 | 0.22 | 1427 | 0.99 | 0.83 | 1.20 | 0.94 | 1.00 |
| Caucasian | rs10735810 | *FokI* | T | 0.37 | 0.37 | 1562 | 1.03 | 0.89 | 1.19 | 0.73 | 1.00 |
| AA | rs2408876 |  | G | 0.46 | 0.48 | 1421 | 0.93 | 0.79 | 1.08 | 0.33 | 1.00 |
| Caucasian | rs2408876 |  | G | 0.41 | 0.43 | 1502 | 0.91 | 0.78 | 1.05 | 0.20 | 0.99 |
| AA | rs2254210 |  | T | 0.34 | 0.33 | 1429 | 1.03 | 0.87 | 1.21 | 0.74 | 1.00 |
| Caucasian | rs2254210 |  | T | 0.35 | 0.32 | 1569 | 1.14 | 0.98 | 1.34 | 0.09 | 0.85 |
| AA | rs11574044 |  | G | 0.27 | 0.25 | 1414 | 1.11 | 0.93 | 1.32 | 0.24 | 1.00 |
| Caucasian | rs11574044 |  | G | 0.15 | 0.17 | 1559 | 0.82 | 0.67 | 0.99 | 0.04 | 0.63 |
| AA | rs11574041 |  | A | 0.09 | 0.10 | 1429 | 0.92 | 0.71 | 1.19 | 0.52 | 1.00 |
| Caucasian | rs11574041 |  | A | 0.00 | 0.00 | 1557 | NA | NA | NA | NA | 1.00 |
| AA | rs2238136 |  | A | 0.10 | 0.10 | 1429 | 1.00 | 0.77 | 1.30 | 0.99 | 1.00 |
| Caucasian | rs2238136 |  | A | 0.27 | 0.28 | 1564 | 0.95 | 0.82 | 1.12 | 0.55 | 1.00 |
| AA | rs2238135 |  | C | 0.32 | 0.33 | 1428 | 0.97 | 0.83 | 1.15 | 0.75 | 1.00 |
| Caucasian | rs2238135 |  | C | 0.25 | 0.27 | 1564 | 0.92 | 0.78 | 1.08 | 0.30 | 1.00 |
| AA | rs2853564 |  | C | 0.12 | 0.12 | 1430 | 1.00 | 0.79 | 1.26 | 0.99 | 1.00 |
| Caucasian | rs2853564 |  | C | 0.39 | 0.35 | 1571 | 1.21 | 1.04 | 1.40 | 0.01 | 0.28 |
| AA | rs2853559 |  | T | 0.17 | 0.16 | 1417 | 1.06 | 0.87 | 1.30 | 0.56 | 1.00 |
| Caucasian | rs2853559 |  | T | 0.39 | 0.35 | 1458 | 1.14 | 0.99 | 1.33 | 0.08 | 0.82 |
| AA | rs11168287 |  | C | 0.27 | 0.29 | 1428 | 0.89 | 0.75 | 1.05 | 0.17 | 1.00 |
| Caucasian | rs11168287 |  | T | 0.50 | 0.47 | 1553 | 1.13 | 0.98 | 1.30 | 0.10 | 0.90 |
| AA | rs4328262 |  | C | 0.30 | 0.32 | 1419 | 0.95 | 0.80 | 1.11 | 0.51 | 1.00 |
| Caucasian | rs4328262 |  | C | 0.42 | 0.45 | 1554 | 0.89 | 0.77 | 1.03 | 0.10 | 0.89 |
| AA | rs4334089 |  | C | 0.38 | 0.38 | 1423 | 1.01 | 0.87 | 1.18 | 0.86 | 1.00 |
| Caucasian | rs4334089 |  | T | 0.29 | 0.28 | 1560 | 1.04 | 0.89 | 1.22 | 0.61 | 1.00 |
| AA | rs3890733 |  | A | 0.15 | 0.14 | 1395 | 1.09 | 0.88 | 1.36 | 0.44 | 1.00 |
| Caucasian | rs3890733 |  | A | 0.31 | 0.29 | 1496 | 1.13 | 0.96 | 1.32 | 0.14 | 0.96 |
| AA | rs7302235 |  | A | 0.49 | 0.50 | 1401 | 0.98 | 0.84 | 1.13 | 0.75 | 1.00 |
| Caucasian | rs7302235 |  | G | 0.28 | 0.28 | 1487 | 1.01 | 0.86 | 1.18 | 0.92 | 1.00 |
| AA | rs7136534 |  | A | 0.10 | 0.11 | 1423 | 0.90 | 0.71 | 1.15 | 0.41 | 1.00 |
| Caucasian | rs7136534 |  | A | 0.27 | 0.26 | 1560 | 1.03 | 0.88 | 1.21 | 0.68 | 1.00 |
| AA | rs11574002 |  | G | 0.04 | 0.03 | 1427 | 1.13 | 0.75 | 1.71 | 0.56 | 1.00 |
| Caucasian | rs11574002 |  | G | 0.00 | 0.00 | 1573 | 1.00 | 0.06 | 16.59 | 1.00 | 1.00 |
| **(B) Rectum** |  |  |  |  |  |  |  |  |  |  |  |
| AA | rs11574141 |  | C | 0.06 | 0.08 | 1048 | 0.74 | 0.46 | 1.19 | 0.21 | 1.00 |
| Caucasian | rs11574141 |  | C | 0.00 | 0.00 | 1202 | 2.75 | 0.60 | 12.54 | 0.19 | 0.99 |
| AA | rs2853563 |  | A | 0.13 | 0.16 | 1042 | 0.83 | 0.59 | 1.15 | 0.26 | 1.00 |
| Caucasian | rs2853563 |  | A | 0.07 | 0.06 | 1186 | 1.23 | 0.87 | 1.75 | 0.24 | 0.99 |
| AA | rs7954412 |  | G | 0.04 | 0.07 | 1047 | 0.65 | 0.38 | 1.11 | 0.12 | 0.99 |
| Caucasian | rs7954412 |  | G | 0.00 | 0.00 | 1200 | 3.80 | 0.33 | 43.43 | 0.28 | 1.00 |
| AA | rs3858733 |  | C | 0.01 | 0.01 | 1048 | 0.52 | 0.12 | 2.26 | 0.38 | 1.00 |
| Caucasian | rs3858733 |  | C | 0.07 | 0.06 | 1197 | 1.11 | 0.78 | 1.58 | 0.57 | 1.00 |
| AA | rs739837 |  | C | 0.40 | 0.42 | 1046 | 0.92 | 0.73 | 1.16 | 0.47 | 1.00 |
| Caucasian | rs739837 |  | C | 0.44 | 0.47 | 1198 | 0.87 | 0.73 | 1.04 | 0.12 | 0.93 |
| AA | rs731236 | *TaqI* | C | 0.32 | 0.30 | 1045 | 1.04 | 0.81 | 1.33 | 0.75 | 1.00 |
| Caucasian | rs731236 | *TaqI* | C | 0.41 | 0.38 | 1187 | 1.13 | 0.95 | 1.34 | 0.17 | 0.98 |
| AA | rs11574114 |  | A | 0.12 | 0.15 | 1047 | 0.82 | 0.58 | 1.15 | 0.25 | 1.00 |
| Caucasian | rs11574114 |  | A | 0.07 | 0.06 | 1196 | 1.16 | 0.82 | 1.65 | 0.40 | 1.00 |
| AA | rs11574110 |  | A | 0.01 | 0.02 | 1034 | 0.90 | 0.34 | 2.39 | 0.84 | 1.00 |
| Caucasian | rs11574110 |  | A | 0.00 | 0.00 | 1189 | NA | NA | NA | NA | 1.00 |
| AA | rs11574105 |  | A | 0.03 | 0.05 | 1042 | 0.58 | 0.30 | 1.13 | 0.11 | 0.99 |
| Caucasian | rs11574105 |  | A | 0.00 | 0.00 | 1195 | 1.60 | 0.35 | 7.26 | 0.54 | 1.00 |
| AA | rs12314197 |  | C | 0.16 | 0.23 | 1048 | 0.67 | 0.50 | 0.91 | 0.01 | 0.29 |
| Caucasian | rs12314197 |  | C | 0.01 | 0.00 | 1201 | 1.70 | 0.51 | 5.68 | 0.39 | 1.00 |
| AA | rs7962898 | *ApaI* | T | 0.42 | 0.35 | 1040 | 1.28 | 1.02 | 1.61 | 0.03 | 0.71 |
| Caucasian | rs7962898 | *ApaI* | C | 0.45 | 0.48 | 1173 | 0.87 | 0.74 | 1.04 | 0.12 | 0.93 |
| AA | rs7967152 |  | A | 0.39 | 0.38 | 1042 | 1.04 | 0.82 | 1.31 | 0.77 | 1.00 |
| Caucasian | rs7967152 |  | A | 0.44 | 0.47 | 1194 | 0.86 | 0.72 | 1.02 | 0.08 | 0.84 |
| AA | rs2239185 |  | C | 0.43 | 0.43 | 1046 | 0.99 | 0.79 | 1.23 | 0.91 | 1.00 |
| Caucasian | rs2239185 |  | C | 0.44 | 0.47 | 1174 | 0.89 | 0.75 | 1.06 | 0.20 | 0.99 |
| AA | rs7971418 |  | C | 0.43 | 0.45 | 1047 | 0.94 | 0.75 | 1.18 | 0.61 | 1.00 |
| Caucasian | rs7971418 |  | C | 0.43 | 0.47 | 1181 | 0.86 | 0.73 | 1.03 | 0.10 | 0.90 |
| AA | rs7975128 | *BsmI* | T | 0.33 | 0.27 | 1046 | 1.23 | 0.97 | 1.56 | 0.09 | 0.95 |
| Caucasian | rs7975128 | *BsmI* | T | 0.42 | 0.39 | 1201 | 1.12 | 0.94 | 1.34 | 0.19 | 0.99 |
| AA | rs11168264 |  | C | 0.18 | 0.24 | 1041 | 0.70 | 0.52 | 0.94 | 0.02 | 0.48 |
| Caucasian | rs11168264 |  | C | 0.00 | 0.01 | 1192 | 0.63 | 0.17 | 2.38 | 0.50 | 1.00 |
| AA | rs7966569 |  | C | 0.05 | 0.04 | 1048 | 1.38 | 0.80 | 2.37 | 0.25 | 1.00 |
| Caucasian | rs7966569 |  | C | 0.00 | 0.00 | 1192 | NA | NA | NA | NA | 1.00 |
| AA | rs7305032 |  | C | 0.30 | 0.30 | 1045 | 1.02 | 0.80 | 1.30 | 0.88 | 1.00 |
| Caucasian | rs7305032 |  | C | 0.39 | 0.42 | 1194 | 0.87 | 0.73 | 1.04 | 0.12 | 0.93 |
| AA | rs11574087 |  | T | 0.02 | 0.03 | 1045 | 0.70 | 0.31 | 1.55 | 0.38 | 1.00 |
| Caucasian | rs11574087 |  | T | 0.00 | 0.00 | 1202 | NA | NA | NA | NA | 1.00 |
| Caucasian | rs11168266 |  | G | 0.39 | 0.43 | 1193 | 0.87 | 0.73 | 1.03 | 0.10 | 0.89 |
| AA | rs11168267 |  | T | 0.08 | 0.07 | 1048 | 1.20 | 0.78 | 1.84 | 0.42 | 1.00 |
| Caucasian | rs11168267 |  | T | 0.10 | 0.09 | 1201 | 1.02 | 0.77 | 1.36 | 0.89 | 1.00 |
| AA | rs11168268 |  | C | 0.37 | 0.36 | 1048 | 1.02 | 0.81 | 1.28 | 0.86 | 1.00 |
| Caucasian | rs11168268 |  | C | 0.39 | 0.43 | 1179 | 0.86 | 0.72 | 1.02 | 0.09 | 0.85 |
| AA | rs12308082 |  | T | 0.06 | 0.09 | 1047 | 0.59 | 0.37 | 0.95 | 0.03 | 0.66 |
| Caucasian | rs12308082 |  | T | 0.00 | 0.00 | 1202 | 1.36 | 0.22 | 8.26 | 0.74 | 1.00 |
| AA | rs2853560 |  | T | 0.03 | 0.04 | 1047 | 0.94 | 0.50 | 1.77 | 0.85 | 1.00 |
| Caucasian | rs2853560 |  | T | 0.00 | 0.00 | 1202 | NA | NA | NA | NA | 1.00 |
| AA | rs2248098 |  | C | 0.54 | 0.49 | 1043 | 1.23 | 0.99 | 1.54 | 0.07 | 0.90 |
| Caucasian | rs2248098 |  | T | 0.47 | 0.50 | 1198 | 0.87 | 0.73 | 1.03 | 0.10 | 0.90 |
| AA | rs987849 |  | C | 0.27 | 0.25 | 1042 | 1.08 | 0.84 | 1.39 | 0.56 | 1.00 |
| Caucasian | rs987849 |  | C | 0.42 | 0.46 | 1156 | 0.85 | 0.72 | 1.01 | 0.06 | 0.74 |
| AA | rs2239182 |  | A | 0.44 | 0.40 | 1046 | 1.17 | 0.93 | 1.47 | 0.19 | 1.00 |
| Caucasian | rs2239182 |  | G | 0.51 | 0.48 | 1181 | 1.13 | 0.96 | 1.34 | 0.14 | 0.96 |
| AA | rs2107301 |  | T | 0.15 | 0.16 | 1048 | 0.95 | 0.69 | 1.30 | 0.75 | 1.00 |
| Caucasian | rs2107301 |  | T | 0.28 | 0.29 | 1175 | 0.95 | 0.79 | 1.15 | 0.59 | 1.00 |
| AA | rs1540339 |  | A | 0.19 | 0.21 | 1042 | 0.87 | 0.65 | 1.16 | 0.35 | 1.00 |
| Caucasian | rs1540339 |  | A | 0.37 | 0.38 | 1195 | 0.95 | 0.79 | 1.13 | 0.54 | 1.00 |
| AA | rs2239179 |  | G | 0.39 | 0.34 | 1038 | 1.20 | 0.95 | 1.51 | 0.13 | 0.99 |
| Caucasian | rs2239179 |  | G | 0.44 | 0.42 | 1144 | 1.10 | 0.93 | 1.30 | 0.28 | 1.00 |
| AA | rs11574070 |  | T | 0.07 | 0.07 | 1038 | 0.99 | 0.63 | 1.57 | 0.98 | 1.00 |
| Caucasian | rs11574070 |  | T | 0.00 | 0.00 | 1197 | 1.40 | 0.12 | 15.78 | 0.79 | 1.00 |
| AA | rs11574065 |  | T | 0.04 | 0.02 | 1047 | 1.92 | 1.02 | 3.60 | 0.04 | 0.79 |
| Caucasian | rs11574065 |  | T | 0.00 | 0.00 | 1202 | 0.00 | 0.00 | inf | 1.00 | 1.00 |
| AA | rs12717991 |  | A | 0.26 | 0.30 | 1048 | 0.85 | 0.66 | 1.09 | 0.20 | 1.00 |
| Caucasian | rs12717991 |  | A | 0.38 | 0.38 | 1194 | 0.97 | 0.82 | 1.16 | 0.77 | 1.00 |
| AA | rs2189480 |  | A | 0.32 | 0.36 | 1034 | 0.82 | 0.64 | 1.05 | 0.11 | 0.98 |
| Caucasian | rs2189480 |  | A | 0.36 | 0.35 | 1182 | 1.04 | 0.87 | 1.24 | 0.68 | 1.00 |
| AA | rs3819545 |  | C | 0.25 | 0.25 | 1044 | 0.97 | 0.74 | 1.26 | 0.81 | 1.00 |
| Caucasian | rs3819545 |  | C | 0.42 | 0.40 | 1184 | 1.06 | 0.89 | 1.26 | 0.49 | 1.00 |
| AA | rs3782905 |  | G | 0.25 | 0.23 | 1043 | 1.11 | 0.86 | 1.44 | 0.41 | 1.00 |
| Caucasian | rs3782905 |  | G | 0.33 | 0.32 | 1199 | 1.05 | 0.88 | 1.26 | 0.57 | 1.00 |
| AA | rs11574050 |  | T | 0.10 | 0.08 | 1047 | 1.25 | 0.85 | 1.83 | 0.25 | 1.00 |
| Caucasian | rs11574050 |  | T | 0.06 | 0.05 | 1201 | 1.01 | 0.70 | 1.48 | 0.94 | 1.00 |
| AA | rs10783218 |  | T | 0.17 | 0.19 | 1042 | 0.87 | 0.64 | 1.18 | 0.36 | 1.00 |
| Caucasian | rs10783218 |  | T | 0.03 | 0.02 | 1194 | 1.48 | 0.91 | 2.40 | 0.12 | 0.93 |
| AA | rs10735810 | *FokI* | T | 0.20 | 0.22 | 1046 | 0.91 | 0.69 | 1.20 | 0.49 | 1.00 |
| Caucasian | rs10735810 | *FokI* | T | 0.35 | 0.37 | 1193 | 0.94 | 0.79 | 1.12 | 0.50 | 1.00 |
| AA | rs2408876 |  | G | 0.49 | 0.48 | 1037 | 1.10 | 0.87 | 1.39 | 0.45 | 1.00 |
| Caucasian | rs2408876 |  | G | 0.43 | 0.43 | 1136 | 0.99 | 0.83 | 1.18 | 0.92 | 1.00 |
| AA | rs2254210 |  | T | 0.30 | 0.33 | 1047 | 0.84 | 0.65 | 1.09 | 0.18 | 1.00 |
| Caucasian | rs2254210 |  | T | 0.34 | 0.32 | 1196 | 1.08 | 0.90 | 1.30 | 0.41 | 1.00 |
| AA | rs11574044 |  | G | 0.27 | 0.25 | 1036 | 1.17 | 0.91 | 1.51 | 0.22 | 1.00 |
| Caucasian | rs11574044 |  | G | 0.18 | 0.17 | 1187 | 1.03 | 0.81 | 1.29 | 0.83 | 1.00 |
| AA | rs11574041 |  | A | 0.07 | 0.10 | 1048 | 0.70 | 0.45 | 1.09 | 0.11 | 0.99 |
| Caucasian | rs11574041 |  | A | 0.00 | 0.00 | 1183 | NA | NA | NA | NA | 1.00 |
| AA | rs2238136 |  | A | 0.08 | 0.10 | 1048 | 0.78 | 0.52 | 1.18 | 0.24 | 1.00 |
| Caucasian | rs2238136 |  | A | 0.27 | 0.28 | 1192 | 0.94 | 0.77 | 1.14 | 0.52 | 1.00 |
| AA | rs2238135 |  | C | 0.27 | 0.33 | 1047 | 0.77 | 0.60 | 0.99 | 0.04 | 0.79 |
| Caucasian | rs2238135 |  | C | 0.26 | 0.27 | 1192 | 0.98 | 0.80 | 1.19 | 0.83 | 1.00 |
| AA | rs2853564 |  | C | 0.14 | 0.12 | 1048 | 1.18 | 0.85 | 1.64 | 0.33 | 1.00 |
| Caucasian | rs2853564 |  | C | 0.35 | 0.35 | 1198 | 1.01 | 0.85 | 1.21 | 0.89 | 1.00 |
| AA | rs2853559 |  | T | 0.19 | 0.16 | 1043 | 1.17 | 0.88 | 1.57 | 0.28 | 1.00 |
| Caucasian | rs2853559 |  | T | 0.34 | 0.35 | 1123 | 0.96 | 0.80 | 1.15 | 0.66 | 1.00 |
| AA | rs11168287 |  | C | 0.30 | 0.29 | 1047 | 1.01 | 0.79 | 1.28 | 0.95 | 1.00 |
| Caucasian | rs11168287 |  | T | 0.45 | 0.47 | 1185 | 0.93 | 0.78 | 1.11 | 0.45 | 1.00 |
| AA | rs4328262 |  | C | 0.35 | 0.32 | 1041 | 1.13 | 0.90 | 1.43 | 0.29 | 1.00 |
| Caucasian | rs4328262 |  | C | 0.46 | 0.45 | 1195 | 1.05 | 0.88 | 1.25 | 0.60 | 1.00 |
| AA | rs4334089 |  | C | 0.42 | 0.38 | 1043 | 1.16 | 0.92 | 1.45 | 0.21 | 1.00 |
| Caucasian | rs4334089 |  | T | 0.26 | 0.28 | 1190 | 0.92 | 0.76 | 1.12 | 0.42 | 1.00 |
| AA | rs3890733 |  | A | 0.16 | 0.14 | 1026 | 1.15 | 0.84 | 1.58 | 0.39 | 1.00 |
| Caucasian | rs3890733 |  | A | 0.29 | 0.29 | 1140 | 1.01 | 0.82 | 1.23 | 0.95 | 1.00 |
| AA | rs7302235 |  | G | 0.44 | 0.50 | 1036 | 0.81 | 0.65 | 1.02 | 0.07 | 0.91 |
| Caucasian | rs7302235 |  | G | 0.26 | 0.28 | 1149 | 0.90 | 0.75 | 1.09 | 0.28 | 1.00 |
| AA | rs7136534 |  | A | 0.11 | 0.11 | 1042 | 0.92 | 0.64 | 1.31 | 0.64 | 1.00 |
| Caucasian | rs7136534 |  | A | 0.24 | 0.26 | 1192 | 0.91 | 0.75 | 1.10 | 0.34 | 1.00 |
| AA | rs11574002 |  | G | 0.03 | 0.03 | 1046 | 1.06 | 0.57 | 2.00 | 0.85 | 1.00 |
| Caucasian | rs11574002 |  | G | 0.00 | 0.00 | 1202 | 6.86 | 0.69 | 67.81 | 0.10 | 0.89 |
| AA, African American; SNP, single nucleotide polymorphism; RFLP, restriction fragment length polymorphism; Freq., frequency; Inds., number of individuals included for each analysis; L95, lower boundary 95% confidence interval; U95, upper boundary 95% confidence interval; NA, not applicable due to monomorphic allele; inf, infinite.  *OR and p-value adjusted for age, gender and West African ancestry (ancestry only adjusted for in AA group). | | | | | | | | | | | |
